# Genetic requirements for repair of lesions caused by single genomic ribonucleotides in S phase

**DOI:** 10.1038/s41467-023-36866-6

**Published:** 2023-03-03

**Authors:** Natalie Schindler, Matthias Tonn, Vanessa Kellner, Jia Jun Fung, Arianna Lockhart, Olga Vydzhak, Thomas Juretschke, Stefanie Möckel, Petra Beli, Anton Khmelinskii, Brian Luke

**Affiliations:** 1 Johannes Gutenberg University Mainz, Institute for Developmental Neurology (IDN), Biozentrum 1, Hanns-Dieter-Hüsch-Weg 15, 55128 Mainz, Germany; 2 Institute of Molecular Biology (IMB), Ackermannweg 4, 55128 Mainz, Germany; 3 Present Address: Department of Biology, New York University, New York, NY USA

**Keywords:** Homologous recombination, Replisome

## Abstract

Single ribonucleoside monophosphates (rNMPs) are transiently present in eukaryotic genomes. The RNase H2-dependent ribonucleotide excision repair (RER) pathway ensures error-free rNMP removal. In some pathological conditions, rNMP removal is impaired. If these rNMPs hydrolyze during, or prior to, S phase, toxic single-ended double-strand breaks (seDSBs) can occur upon an encounter with replication forks. How such rNMP-derived seDSB lesions are repaired is unclear. We expressed a cell cycle phase restricted allele of RNase H2 to nick at rNMPs in S phase and study their repair. Although Top1 is dispensable, the *RAD52* epistasis group and Rtt101^Mms1-Mms22^ dependent ubiquitylation of histone H3 become essential for rNMP-derived lesion tolerance. Consistently, loss of Rtt101^Mms1-Mms22^ combined with RNase H2 dysfunction leads to compromised cellular fitness. We refer to this repair pathway as nick lesion repair (NLR). The NLR genetic network may have important implications in the context of human pathologies.

## Abstract (web summary)

RNase H2 removes mutagenic rNMPs from genomic DNA. The authors demonstrate how nicked rNMPs are repaired when they are encountered in S phase. This study unveils genetic interactions that could potentially be exploited in RNase H2-deficient pathologies.

## Introduction

Single ribonucleoside monophosphates (rNMPs) are present in the genomic DNA (gDNA) of yeast and human cells. The budding yeast *Saccharomyces cerevisiae* incorporates about 10,000 rNMPs into the genome per cell cycle^1^. DNA polymerases incorporate rNMPs during the DNA replication process. The double-stranded context of gDNA does not permit the reduction of the 2′-hydroxyl (2′-OH) group of the misincorporated rNMP, hence its presence can lead to single-strand DNA (ssDNA) breaks (nicks) of the sugar-phosphate backbone that result in replication fork collapse and the formation of single-ended double-strand breaks (seDSBs) in S phase. In addition, genomic rNMPs themselves hinder the passage of DNA polymerases and cause replication stress; a phenotype conserved from yeast to human cells^1–6^. Therefore, it is critical to remove rNMPs in a timely manner to prevent rNMP-derived genomic instability.

RNase H2 is the central ribonucleotide excision repair (RER) enzyme in yeast and mammalian cells^7^. The majority of rNMPs that have been incorporated into nascent gDNA during DNA replication in S phase can be removed in the subsequent G2 phase^8^. When RER fails, topoisomerase 1 (Top1) can process genomic rNMPs. Top1 action at rNMPs is however associated with genomic instability due to error-prone branching in the Top1 pathway^9,10^. Even in the presence of RER, Top1 nicks some rNMPs^11^, but the precise interplay between RNase H2 and Top1 remains to be elucidated. Nonetheless, timely elimination of genomic rNMPs is crucial, and defective RER is associated with human diseases such as chronic lymphocytic leukemia and prostate cancer^6^.

RNase H2 is a trimeric enzyme in yeast (*RNH201, RNH202, RNH203*) and mammalian cells (RNASEH2A, RNASEH2B, RNASEH2C)^7^. We previously engineered cell cycle regulated alleles for the *RNH202* gene to restrict expression of the enzyme to either the S or G2 phase of the cell cycle^8^. In addition to the finding that expression of *RNH202* exclusively in G2 was sufficient to suppress RER defects, we observed an unexpected fitness defect when RNase H2 activity was restricted to S phase^8^. Yeast with S phase expressed *RNH202* (*S-RNH202-TAP, referred to as S-RNH202*) experienced toxicity caused by nicking of the gDNA and relied on the homology-directed repair (HDR) factor Rad52 for survival^8^. The decreased fitness of the *S-RNH202* strain was strongly exacerbated in presence of the *pol2-M644G* allele, a Polymerase ε (Pol ε) mutant that incorporates 10-fold more rNMPs^1,12^. Notably, the *S-RNH202* phenotype was independent of Top1 activity suggesting that the RNase H2-mediated nicking of rNMPs in the S phase can cause toxic seDSBs during replication.

Surprisingly, rNMP accumulation can be tolerated in the absence of both RER and Top1 pathways. This is evidenced by the viability of budding yeast lacking Top1 and expressing an allele of RNase H2 that is deficient in RER (but proficient in R-loop removal)^13^. This implies that cells can either tolerate the presence of replication stress and DNA damage from rNMPs, or that there might be other rNMP lesion repair pathways that act independently of RNase H2 and Top1 (discussed in ref. ^14^).

In this study, we set out to get a better understanding of how rNMP-induced DNA lesions are repaired, when the nicking occurs during, or prior to, DNA replication. We used the *S-RNH202* allele as a molecular tool to promote nicking of genomic rNMPs in S phase^8^. Using synthetic genetic array (SGA) technology^15^, we demonstrate that the *RAD52* HDR epistasis group, the histone remodelers Asf1 and Rtt109, the STR (Sgs1-Top3-Rmi) complex, the Mus81-Mms4 resolvase, and the E3-Ubiquitin ligase complex Rtt101, Mms1, and MmsS22 are all required for the tolerance of rNMP-derived nicks during S phase. These factors comprise a Top1-independent, rNMP-derived nick lesion repair (NLR) pathway. We also found that histone H3 ubiquitylation by the replisome-associated Rtt101^Mms1-Mms22^ complex is critical for NLR in high rNMP conditions, pointing to a role for chromatin remodelling in NLR.

We summarize our genetic data in a model that likely represents the molecular processes in the NLR repair pathway. When a replication fork runs into an rNMP-derived strand nick, whether it be on the leading or lagging strand, a seDSB is formed. Rtt101-dependent post-translational modifications at chromatin and perhaps elsewhere then promote HDR. The STR and Mus81-Mms4 complexes are needed to provide resolution of the recombination intermediates. Importantly, we also report a negative genetic interaction between *RTT101* and the complete loss of RNase H2, which becomes synthetic lethal when the genomic rNMP load increases. These data in yeast may provide therapeutic insights and alternatives for human cancer treatment in genetic contexts where RNase H2 is dysfunctional such as RER-deficient cancers^6^.

## Results

### Genetic screen identifies a network required for rNMP-derived lesion tolerance in S phase

We employ the *S-RNH202-TAP* allele (from here on referred as *S-RNH202*) as a tool to endogenously nick genomic rNMPs in S phase. Restricting the expression of RNase H2 to S phase also results in the accumulation of genomic rNMPs, which supports the notion that the bulk of RER occurs outside of the S phase. Indeed, the rNMP load is similar in *S-RNH202* as it is in RER-deficient strains^8^. Consistently, the *S-RNH202* allele has a similar rate of mutagenesis as the RNase H2 deletion (*rnh202*Δ) in the presence of the *pol2-M644G* allele, a Polymerase ε (Pol ε) mutant that increases the rNMP load by 10-fold^12^ (Supplementary Fig. 1A). RNase H2 deletion and *S-RNH202* expressing strains share not only the same amount of genomic rNMPs, and the same mutagenesis rate but also are both highly sensitive towards hydroxyurea (HU) (in the *pol2-M644G* background) (Supplementary Fig. 1B). Methyl methane sulfonate (MMS) stabilizes R-loops which are potentially toxic RNA-DNA hybrids that are removed by RNase H1 and RNase H2^8^. In the presence of MMS, the *rnh1*Δ *rnh201*Δ double mutant and *rnh1*Δ *S-RNH202* double mutant are inviable (Supplementary Fig. 1C). Therefore, both canonical RNase H2 functions, R-loop removal and RER, likely occur outside of S phase. Taken together, these data suggest that employing the *S-RNH202* allele as an enzymatic tool to endogenously nick genomic rNMPs recapitulates many phenotypes of an RNase H2 deletion, and suggests that many problems associated with loss of RER are due to rNMP nicking during DNA replication. Therefore, the *S-RNH202* allele is relevant both in terms of understanding rNMP repair during RER deficiency (rNMP hydrolysis in S phase) and in canonical RER, when RNase H2 nicked rNMPs are not repaired in a timely manner and are encountered in the following S phase.

**Fig. 1:**
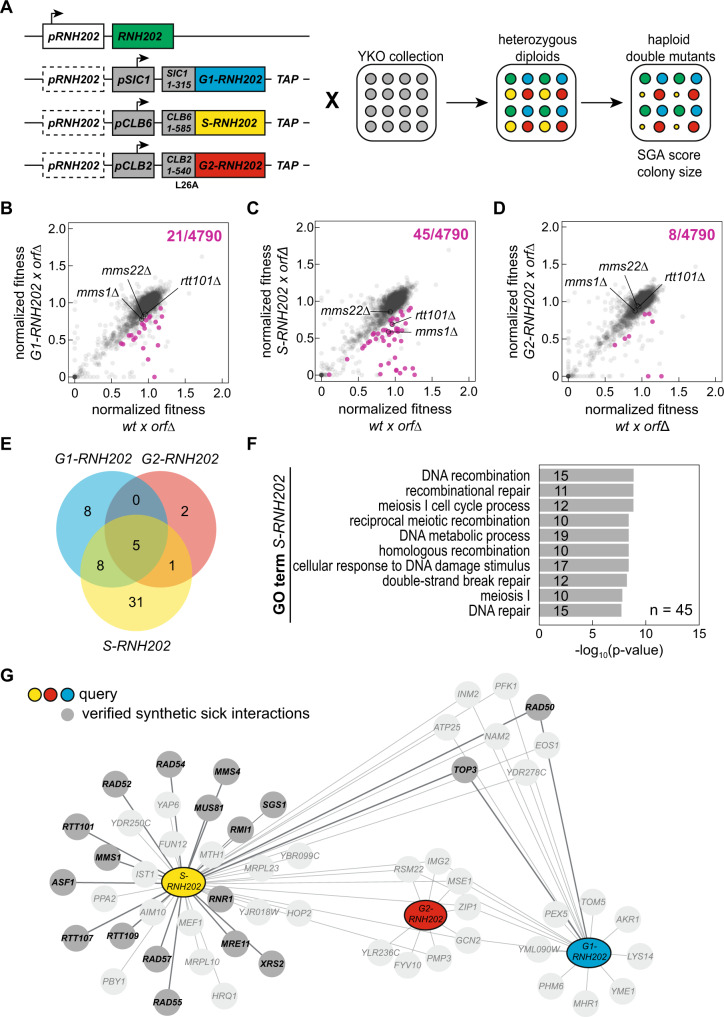
SGA screen identifies network of genes required for rNMP-derived lesion tolerance in S phase. **A** For the SGA analysis, the illustrated three query strains (*G1-RNH202*, *S-RNH202*, *and G2-RNH202*) were crossed to the non-essential yeast knockout (YKO) collection. The heterozygous diploids were sporulated and fitness of the resulting haploid double mutants was scored based on their colony size. The outcome was compared to the corresponding scores from the wild type (*RNH202*) cross. For each genotype, four replicates per strain were generated and analyzed. Scheme was created using Adobe Illustrator. **B**–**D** Scatter plots of normalized double mutant fitness for the queries compared to wild type (wt). The three queries with cell cycle *RNH202* alleles compared to the wild type control (wt). Each data point represents a single mutant in the YKO collection. Significant synthetic sick interactions (fitness query x *orf*Δ wt x *orf*Δ <0.8, *p* < 0.05 in a t-test, corrected for multiple testing using the Benjamini-Hochberg method) are highlighted in magenta. Crosses with *mms1*Δ, *mms22*Δ and *rtt101*Δ mutants are indicated. Top right, total number of significant synthetic sick interactions. **E** Venn diagram of the number of synthetic sick interactions for the three queries with the cell cycle restricted *RNH202* alleles. **F** GO term enrichment analysis for synthetic sick interactions of the *S-RNH202* query. Only Biological Process GO terms are shown, top 10 terms by *p*-value in a hypergeometric test. **G** Network summary of the synthetic sick interactions for the three queries with the cell cycle *RNH202* alleles. Genes mapped to the GO term „DNA metabolic process” are highlighted in grey. We could exclude genes with linkage to the *rnh202*Δ locus, and manual tetrad dissection identified false positives (Supplementary Data 1). These false positive hits were excluded from the network (faint appearance in the scheme).

To identify factors involved in repair of rNMP-derived lesions occurring in S phase we performed a synthetic genetic array (SGA) analysis with cell cycle restricted alleles of *RNH202* in the budding yeast *Saccharomyces cerevisiae* (Fig. 1A). We generated G1-, S-, and G2-restricted alleles of *RNH202* in the query background (Supplementary Fig. 1D, E). Then, we crossed the three queries along with the wild type control to the haploid yeast knockout collection (YKO) of non-essential genes. We derived haploid double mutants and determined their fitness by measuring colony size (Fig. 1A). We compared the hits of each *RNH202* cell cycle allele with the wild type *RNH202* control to identify allele-specific genetic interactions (representative examples Supplementary Fig. 1F–H). Out of 4790 gene knockouts included in the screen, we identified 21 synthetic sick interactions for the *G1-RNH202* allele (Fig. 1B), 45 for the *S-RNH202* allele (Fig. 1C), and eight for the *G2-RNH202* allele (Fig. 1D). Of those hits, five genes were essential to support normal colony size among all three alleles (Fig. 1E). Gene Ontology (GO) revealed that the GO processes related to “DNA recombination” and “DNA repair” were enriched among the 45 synthetic sick interactions of the *S-RNH202* allele (Fig. 1F). This is in line with our previous finding that the HDR factor Rad52 is essential in the *S-RNH202* genetic background^8^. We tested all candidates by manual tetrad dissection and curated the genetic interaction network accordingly (Supplementary Data 1, Supplementary Fig. 1I, J, and Fig. 1G). Among the synthetic sick interactions unique to the *S-RNH202* allele, we identified *RAD52* epistasis group genes (*RAD52, RAD54, RAD55, RAD57*) consistent with our previous report^8^, the *MUS81-MMS4* nuclease complex, the *SGS1-TOP3*-*RMI1* (STR) helicase complex, the *MRE11-XRS2-RAD50* (MRX) nuclease complex, the nucleosome assembly factors *RTT109 and ASF1* and the *RTT101*^*MMS1*^ ubiquitin ligase (Supplementary Fig. 1I and Fig. 1G). None of the G2-specific synthetic sick interactions was confirmed by manual tetrad dissection and thus were false-positives, consistent with canonical RER occurring in this phase of the cell cycle (Supplementary Fig. 1J). Interestingly, translesion synthesis appears to not play a role for maintaining *S-RNH202* fitness (Supplementary Fig. 1K).

Surprisingly, only two hits were confirmed with the *G1-RNH202* allele, both involved in the HDR pathway. The G1 allele is the least tightly regulated of all *RNH202* alleles (Supplementary Fig. 1E). To exclude that the complementation of *G1-RNH202* was due to a weak expression of the G1 allele into S phase we performed synchronization experiments combined with induced-expression of RNase H2 only in G1 phase. These unpublished data support the spotting in Supplementary Fig. 1B and will be part of the future characterizing of RNase H2 activity in the G1 phase. In summary, the results with the *G1-RNH202* allele indicate that RNase H2 initiated rNMP-repair may also take place in G1 phase. However, we could envisage that HDR is needed to an extent, in the case that nicked rNMPs from G1 are passed into the following S phase where these nicks again meet the replisome and ultimately would form seDSBs.

A recent study identified genetic interactions when RNase H2 is absent, and included both non-essential gene deletions as well as essential mutants from the temperature-sensitive yeast collection^16^. We identified seven genetic interactions that are shared between RER-deficiency (*rnh201*Δ) and S phase restricted RNase H2 (*S-RNH202*) (Supplementary Fig. 2A, B). Plotting the genetic interaction String network of the complete hit list from the Chang et al. study allowed us to identify a gene cluster comprising eleven genes, including the MRX complex (*MRX*-*RAD50*-*XRS2*)(Supplementary Fig. 2C, D, *red circle*). Of those eleven genes of the “MRX cluster”, seven genes constitute the identified non-essential shared hits (Supplementary Fig. 2A, B). We could validate the other four genes in the MRX cluster as true synthetic sick interactions with *S-RNH202* (Supplementary Fig. 2E). In conclusion, RER-deficiency and *S-RNH202* require the same genetic network to maintain fitness, i.e., the HDR pathway. The *S-RNH202* expression renders cells more dependent on HDR, as the accumulated rNMPs are being actively hydrolyzed by RNase H2 activity, whereas the rNMPs in the RNase H2 deletion strains subject to spontaneous hydrolysis, a less frequent occurrence.

The SGA screen and subsequent validation identified 17 candidate genes linked to DNA metabolic processes, including DNA resection, HDR, and repair intermediate resolution, that may be involved in repair of rNMP-derived gDNA lesions in S phase.

### Rtt101 acts in a genetic pathway with Rad51 to promote rNMP repair

**Fig. 2:**
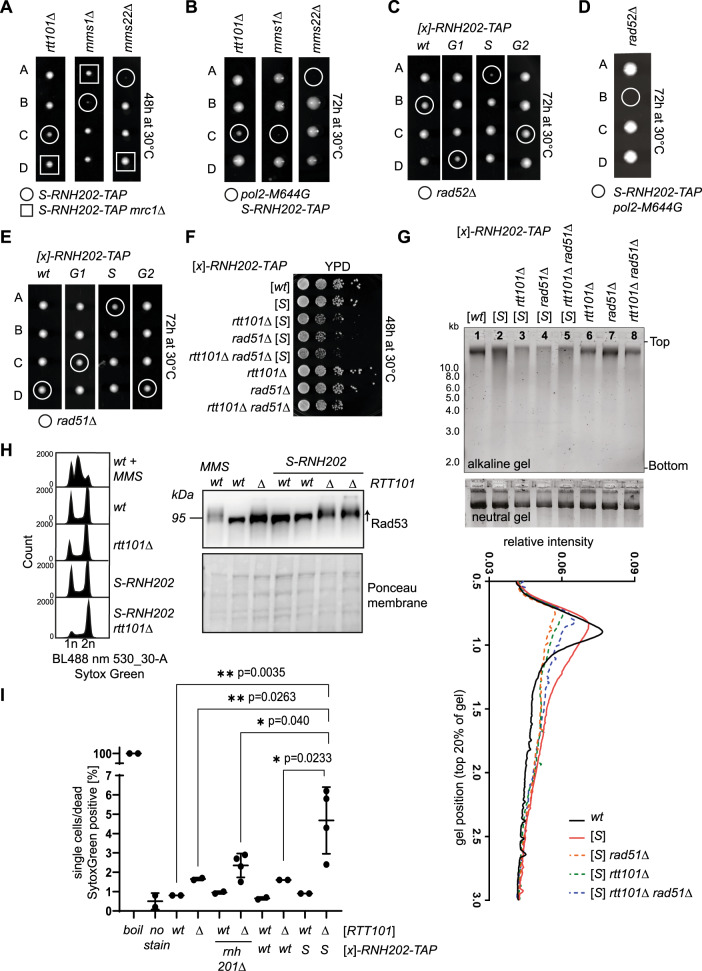
Rtt101, histone modifiers and HDR factors promote cell viability when genomic rNMPs are hydrolyzed in S phase. **A** Tetrads from Rtt101^Mms1-Mms22^ complex-deficient diploid strains in combination with *S-RNH202-TAP* revealed smaller colony sizes (colonies in circles). The genetic suppressor of *rtt101*Δ, *mrc1*Δ is sufficient to fully reverse the growth phenotype^19^ (colonies in squares). **B** Tetrads from dissections of Rtt101^Mms1-Mms22^ complex-deficient diploid strains in the *S-RNH202-TAP pol2-M644G* genetic background augmented the sickness. The *rtt101*Δ *S-RNH202-TAP pol2-M644G* lethality was less penetrant compared to the *mms1*Δ and *mms22*Δ mutants, but after propagating, these small colonies were mostly inviable or acquired suppressor mutations. **C** Tetrad dissections of *rad52*Δ with *RNH202-TAP* cell cycle alleles demonstrates that the *S-RNH202-TAP rad52*Δ double mutant is inviable. **D** The phenotype from (**C**) was exacerbated when increasing the rNMP load (*pol2-M644G*). **E** The tetrads for *RAD51* deletion with all cell cycle alleles of *RNH202* shows that the *S-RNH202 rad51*Δ double mutant is sick but viable. **F** Spot assay shows epistasis between *S-RNH202-TAP rtt101*Δ and *S-RNH202-TAP rad51*Δ double mutants and the *S-RNH202-TAP rtt101*Δ *rad51*Δ triple mutant. **G** Alkaline gel electrophoresis of the genotypes used in F) showed epistasis in terms of gDNA fragmentation between *S-RNH202-TAP rtt101*Δ and *S-RNH202-TAP rad51*Δ double mutant and the *S-RNH202-TAP rtt101*Δ *rad51*Δ triple mutant (compare lanes 3, 4, 5). The neutral gel serves as control for gDNA purity and integrity. Two alkaline gels revealed the same result. **H** Western blot analysis of checkpoint status by phospho-shift analysis of Rad53. The DNA profiles showed a 2n peak accumulation in line with the activated Rad53 checkpoint. **I** Viability analysis of exponential cultures stained with SYTOX Green shown as Scatter dot blot (line = mean, error bars = SD). “Boil” is the boiled positive control representing 100% dead hence SYTOX green positive cells. The unstained wild type sample serves as background control. Statistical analysis with GraphPadPrism8 (multiple comparison ANOVA test), *n* = 2 for all samples except *rnh201*Δ *rtt101*Δ and *S-RNH202-TAP rtt101*Δ n = 4 samples. Source data are provided as a Source Data file.

Genetic evidence points to a crucial role of the Rtt101^Mms1-Mms22^ ubiquitin ligase complex in the regulation of DNA repair and chromatin establishment^17–21^. These studies addressed the role of Rtt101 in the presence of exogenously induced DNA damage such as the Top1 poison camptothecin (CPT), the alkylating agent methylmethanesulfonate (MMS), or the ribonucleotide reductase inhibitor hydroxyurea (HU). Here, we found that Rtt101and the adaptor subunit Mms1 are also required when endogenous DNA lesions at genomic rNMP arise in S phase (Fig. 1G). *MMS22*, a member of the complex, was not a hit in the screen but was manually confirmed thereafter (Fig. 2A).

Consistent with the entire Rtt101^Mms1-Mms22^ complex being important for rNMP tolerance, the individual deletions of *RTT101*, *MMS1*, and *MMS22* are all compromised for growth in combination with *S-RNH202* (Fig. 2A). While colony growth is mildly affected in *rtt101*Δ *S-RNH202*, the deletion of *MMS1* and *MMS22* results in stronger effects (Fig. 2A, circled colonies). Increasing the rNMP load 10-fold using the *pol2-M644G* allele augments the synthetic sickness of *S-RNH202* expression in Rtt101 complex mutants (Fig. 2B). Although small spores form initially, they eventually become enviable, indicating the presence of severe genomic instability in these strains.

The RNase H2 enzyme contains a C-terminal PIP box motif that mediates binding to proliferating cell nuclear antigen (PCNA), which enhances RNase H2 processivity in vitro^22^. The PIP box motif in RNase H2 is not required to produce the synthetic sick genetic interaction between *rtt101*Δ and *S-RNH202* (Supplementary Fig. 2F, G), suggesting PIP box independent toxicity.

The fork protection protein and checkpoint regulator Mrc1 is a genetic suppressor of MMS and CPT-sensitivity in *rtt101*Δ cells^19^. Noteworthy, *mrc1*Δ was also sufficient to rescue the growth of Rtt101^Mms1-Mms22^ deficient *S-RNH202* strains (Fig. 2A). We are following up the underlying mechanism as to why *mrc1*Δ rescues this phenotype, which will be described elsewhere.

We tested the notion that Top1 may be responsible for the synthetic sick interaction of *S-RNH202* and *rtt101*Δ. Since the *rtt101*Δ *S-RNH202 pol2-M644G* mutant is inviable (Fig. 2B), we switched to HU instead of *pol2-M644G* to modulate the genomic rNMP load. HU has been previously shown to increase the genomic rNMP load in yeast and mammalian cells^23,24^. As expected, in the presence of HU, the *S-RNH202* allele was synthetic sick with *rtt101*Δ (Supplementary Fig. 3A). Although the Top1-mediated RER-backup pathway can be mutagenic and accounts for cellular toxicity in absence of RNase H2^10^, the synthetic sickness of *rtt101*Δ *S-RNH202* was Top1-independent (Supplementary Fig. 3B). Moreover, the overexpression of RNase H1 did not improve growth in the *rtt101*Δ *S-RNH202* strains, suggesting that the toxicity is R-loop independent (Supplementary Fig. 3C). Finally, we combined the *S-RNH202* allele with the RER-deficient *rnh201-RED* allele, a separation of function mutant of RNase H2 (^13^, reviewed in^25^), that is RER defective, but can still remove R-loops, to show that hydrolyzed rNMPs in S phase require Rtt101 (Supplementary Fig. 3D, E). The spot assay and the liquid growth assay data solidify that the *rtt101*Δ *S-RNH202* double mutant has reduced viability due to the RNase H2 ribonucleotide excision activity in S phase as the *rtt101*Δ *S-RNH202 RNH201* mutant is sicker and has longer population doubling time compared to the *rtt101*Δ *S-RNH202 rnh201*Δ mutant and the *rtt101*Δ *S-RNH202 rnh201-RED* strain (Supplementary Fig. 3D, E). Together, we showed that the role of Rtt101 in cells with high rNMP load and RNase H2 dysfunction is independent of Top1 and R-loops and dependent on the hydrolysis of genomic rNMPs by, for example the S-phase restricted RNase H2 allele. To test if Rtt101 and other HDR genes promote the repair of genomic rNMPs and their lesions, we performed genetic epistasis experiments combined with alkaline gel electrophoresis to monitor the genomic rNMP abundance in the presence and absence of the putative repair pathway. As previously demonstrated, the *RAD52* gene becomes essential when rNMPs hydrolyze enzymatically through *S-RNH202* (Fig. 2C). This phenotype is exacerbated when the rNMP load is increased through *pol2-M466G* expression (Fig. 2D). Expression of *G1-RNH202* also slightly affects the growth of *rad52*Δ cells, which again suggests that RNase H2 may be acting in G1, but some unrepaired nicks are carried into S phase (Fig. 2C). Due to the lethality of *RAD52* deletions in the *S-RNH202* genetic background, we could not perform genetic epistasis experiments with loss of *RTT101*. The *rad51*Δ *S-RNH202* double mutant, however, is growth impaired, yet viable (Fig. 2E). Hence, we performed epistasis experiments comparing the loss of *RTT101* and *RAD51* and both genes together in the *S-RNH202* background (Fig. 2F). The *rtt101*Δ *rad51*Δ *S-RNH202* triple mutant is not additive, suggesting that *RTT101* and *RAD51* may function in the same genetic pathway of rNMP-derived nick repair in S phase (Fig. 2F). We employed alkaline gel electrophoresis to visualize the genomic rNMP load in *rtt101*Δ and *rad51*Δ strains in the presence of increased S phase rNMP-nicking (*S-RNH202*). In addition to rNMP-hydrolysis activity in S phase, the *S-RNH202* strain accumulates rNMPs (Fig. 2G, lane 1 compared to lane 2). Strikingly, the loss of either *RTT101* or *RAD51* alone, and in combination, resulted in higher DNA fragmentation in alkaline conditions and loss of the prominent genomic DNA band, indicative of fragmented genomic DNA, hence lack of rNMP repair (Fig. 2G, lanes 3, 4, 5 compared to 2, and quantification graph below). The *rtt101*Δ strains show basal checkpoint activation visualized by phospho-Rad53 analysis (Fig. 2H). In line with the elevated rNMP load and non-repaired DNA damage leading to impaired viability, the *rtt101*Δ *S-RNH202* mutants have fully activated the Rad53-checkpoint (Fig. 2H). In addition, we observed 4% cell death in that population without further challenge (Fig. 2I). This supports the idea of a repair pathway as loss of the repair factors, Rtt101 and Rad51, result in a repair defect accompanied by an activated DNA damage checkpoint, hence the failure to efficiently remove rNMPs. Epistasis between *rtt101*Δ and *rad51*Δ in *S-RNH202*-cells was also confirmed by DNA damage checkpoint activation (Supplementary Fig. 3F, G) and by population doubling time measurement in liquid cultures (Supplementary Fig. 3H). Together, these data demonstrate that the Rtt101 complex works together with the recombination machinery to repair rNMPs, and not R-loops, that get nicked in the S phase.

### Rtt101 becomes essential in S phase to overcome Top1-independent rNMP-derived toxicity

**Fig. 3:**
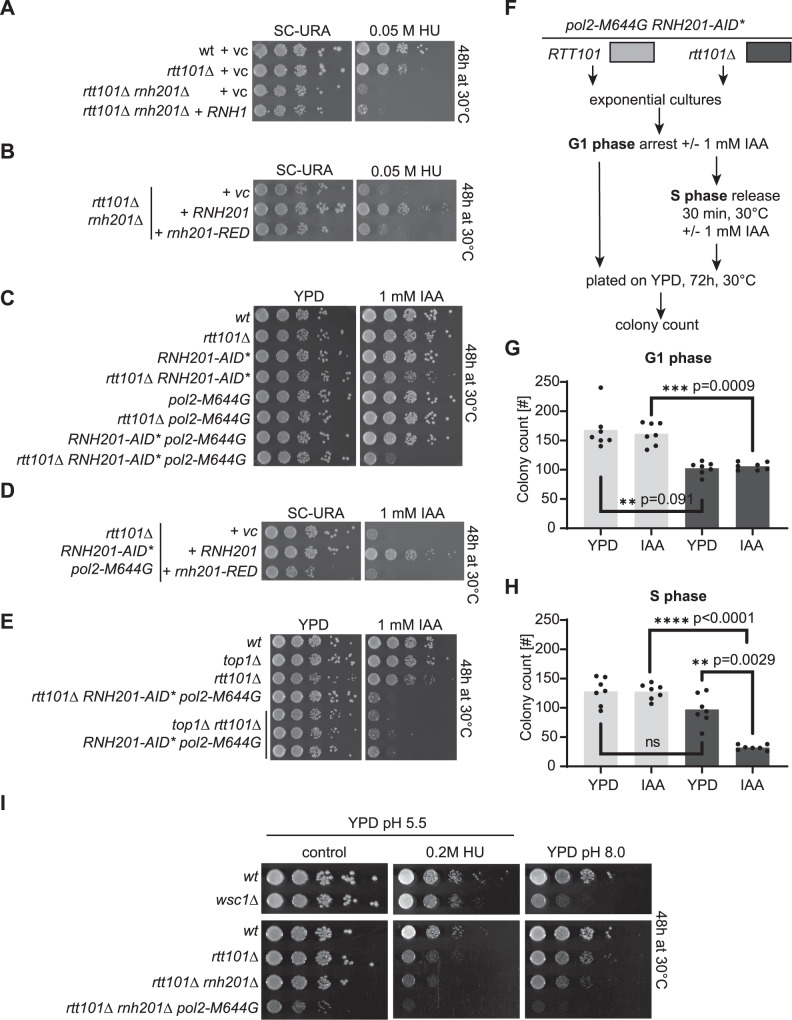
Rtt101 becomes essential in S phase in a Top1-independent manner to overcome rNMP-derived toxicity. **A** The *rtt101*Δ *rnh201*Δ double mutant is synthetic lethal in the presence of HU (hydroxyurea). Transformation with *RNH1* did not affect the growth of the double mutant on HU-containing agar plates. **B** Transformation with *RNH201* did rescue growth of the *rtt101*Δ *rnh201*Δ double mutant on HU plates, while the RER-deficient separation-of-function *rnh201-RED* mutant had no effect. Two independent transformants of each genotype showed the same result. **C** Depletion of RNH201-AID* in the presence of IAA (auxin) resulted in a synthetic sick growth phenotype with *rtt101*Δ, which was amplified into a synthetic lethal phenotype when combined with *pol2-M644G*. **D** Complementation of the *pol2-M644G rtt101*Δ RNH201-AID* triple mutant with wild type *RNH201* rescued growth on auxin plates, while the *rnh201-RED* mutant did have no effect. **E** The synthetic lethality of the *pol2-M644G rtt101*Δ RNH201-AID* triple mutant on auxin plates was Top1-independent. Three independent strains from separate tetrads were spotted to confirm Top1-independence of the observed phenotype. **F**–**H** Liquid cultures with the indicated genotypes were synchronized with α-factor in the G1 phase in the presence of IAA to deplete RNH201-AID*. The arrested cultures were either directly plated on YPD agar plates (colony count panel **G**), or released into the S phase in the presence of IAA, followed by plating on YPD agar plates (colony count panel H). Scatter dot blot with bar at mean from *n* = 7 plate counts per genotype and condition. Statistics were performed with GraphPad Prism8 (multiple comparison ANOVA). **I** The spot assay with *rtt101*Δ strains in the RER-deficient *rnh201*Δ and the rNMP accumulating *pol2-M644G* background revealed that alkalization of the YPD agar was sufficient to phenocopy the synthetic sick growth defects seen in the presence of HU. Source data are provided as a Source Data file.

We have demonstrated that the *S-RNH202* allele is very similar to the RNase H2 deletion (Supplementary Fig. 1A–C), hence we predicted that *RTT101* would also play an important role in the S phase repair of hydrolyzed rNMPs in RER-deficient strains. Indeed, the *rtt101*Δ *rnh201*Δ double mutants were sensitive to HU as compared to the respective single mutants (Fig. 3A). Importantly, *RNH1* overexpression, which reduces R-loop levels, did not rescue the *rtt101*Δ *rnh201*Δ viability defect in the presence of HU (Fig. 3A), suggesting that R-loops may not be responsible for the growth defects. As RNase H2 has a dual role in RNA-DNA hybrid removal, and participates in R-loop removal^7,26^, we again employed the *rnh201-RED* allele that retains R-loop removal activity but fully lacks RER-activity^13^. We found that the RER-proficient *RNH201* wild type allele could rescue the growth defect of *rtt101*Δ *rnh201*Δ mutants in the presence of HU; however, strains expressing the *rnh201-RED* allele were as sick as the vector control (Fig. 3B). This confirmed that persisting genomic rNMPs are the underlying cause of the slow growth in *rtt101*Δ *rnh201*Δ cells (Fig. 3A, B). The *rtt101*Δ *rnh201*Δ *pol2-M644G* triple mutant was genetically unstable, therefore we employed an RNH201-AID* auxin-inducible degron^27^, to highly reduce RNase H2 activity in the presence of auxin (Supplementary Fig. 4A, B). Similar to the *rtt101*Δ *rnh201*Δ double mutant, *rtt101*Δ RNH201-AID* cells presented a mild growth defect upon exposure to auxin (Fig. 3C). Upon addition of the *pol2-M644G* allele to increase the genomic rNMP load, the *rtt101*Δ RNH201-AID* pol2-M644G triple mutant was inviable in the presence of auxin (Fig. 3C). The *rnh201-RED* allele could not rescue the synthetic lethality of the *rtt101*Δ RNH201-AID* pol2-M644G triple mutants in the presence of auxin (Fig. 3D). The deletion of either *MMS1* or *MMS22* showed similar genetic interactions with *RNase H2* impairment, suggestive of the entire E3 ubiquitin ligase complex being required to tolerate increased rNMP levels (Supplementary Figure 4C). As with the *S-RNH202 allele*, we tested whether Top1-processing of ribonucleotides was responsible for the phenotype of *rtt101*Δ RNH201-AID* pol2-M644G cells. In line with the *S-RNH202 allele* (Supplementary Fig. 3B), the deletion of *TOP1* did not rescue the viability of *rtt101*Δ RNH201-AID* pol2-M644G in the presence of auxin (Fig. 3E). The inability of *top1*Δ to rescue the phenotype was consistent for the entire Rtt101^Mms1-Mms22^ complex (Supplementary Figure 4D). As we previously demonstrated that *RTT101* acts in the same pathway as HDR for survival with *S-RNH202* expression, we also expected that defective RER would lead to a fitness disadvantage when HDR was inactive. To this end, we observed that the loss of *RAD52* was defective for growth in the presence of the *rnh201-RED* allele (Supplementary Fig. 4E*)*. The viability of a RER-deficient *pol2-M644G* strain fully relied on the presence of *RAD52*, furthermore indicating that the lesion potential correlates directly with the amount of rNMPs (Supplementary Fig. 4F). Together, these results are consistent with an Rtt101-mediated HDR being required to repair nicked rNMPs in S phase, independent of Top1 mediated cleavage.

The Rtt101^Mms1-Mms22^ ubiquitin ligase complex is associated with the replisome during S phase^19^ and becomes essential when rNMPs are hydrolyzed in S phase by *S-RNH202* (Figs. 1, 2). We wanted to test if rNMP-derived damage in a single S phase requires the immediate activity of Rtt101^Mms1-Mms22^. Therefore, we performed a colony formation assay to assess cell viability when rNMP removal is prevented either in the G1 phase or during S phase (Fig. 3F–H and Supplementary Fig. 4G, H). We arrested RNH201-AID* pol2-M644G and *rtt101*Δ RNH201-AID* pol2-M644G cultures in the G1 phase in the presence of auxin to degrade Rnh201 and prevent RNase H2 activity. To assess the toxicity of rNMP accumulation in G1 phase, we plated the cultures directly on rich medium and quantified the number of colonies formed (Fig. 3G). Alternatively, the synchronized cultures were released from the G1 arrest into the S phase still in the presence of auxin to abolish RNase H2 activity during S phase entry and progression. S phase cultures were also plated on rich medium, thereby allowing the re-accumulation of RNase H2 (Fig. 3H). We monitored the cell cycle phases of the cultures by flow cytometry (Supplementary Fig. 4H). We observed an overall 20% viability reduction in the *rtt101*Δ background (Fig. 3G, H). RER-deficiency did not affect the cell viability during G1 phase (Fig. 3G, compare dark grey columns). However, in the absence of Rtt101 there was a 70% reduction in cell viability when RER-deficient cells progressed through S phase (Fig. 3H, compare dark grey columns). Therefore, non-repaired rNMPs are only toxic in *rtt101*Δ cells in the S phase of the cell cycle, and not in G1.

The 2′-hydroxyl group renders rNMPs susceptible to spontaneously hydrolyze the phosphodiester backbone compared to the more stable and resistant DNA deoxy sugars. Since this hydrolysis reaction is more likely in a basic environment, we assumed that growth in alkaline conditions may increase the likelihood that hydrolysis at genomic rNMPs will occur. Alkaline conditions were therefore expected to impact the growth of *rtt101*Δ strains similar as the presence of hydroxyurea or the absence of RER. *Saccharomyces cerevisiae* media (YPD) has pH5.5 and therefore is mildly acidic. We increased the pH of solid agar medium to pH8.0. We confirmed alkaline pH8.0 in agar plates by using the *wsc1*Δ strain that renders cells sensitive to alkali pH stress^28^ (Fig. 3I). The *rtt101*Δ strain was mildly sensitive to pH8.0 whereas *rtt101*Δ *rnh201*Δ cells were highly sensitive to alkaline conditions (Fig. 3I). The unstable *rtt101*Δ *rnh201*Δ *pol2-M644G* triple mutant was fully inviable on pH8.0 (Fig. 3I).

So far we utilized hydroxyurea (HU) or the leading strand mutator *pol2-M644G* to elevate genomic rNMP levels. As the SGA screen (Fig. 1) was performed in presence of the wild type polymerase alleles *POL3* and *POL2* we anticipate that the rNMP-derived lesions in the *S-RNH202* strain should occur randomly in the genome. Indeed, the *pol3-L612M* allele recapitulated the negative synthetic effects in RER-deficient and S phase restricted RNase H2 conditions in the absence of *RTT101* (Supplementary Fig. 5A–C). Compared to *pol2-M644G, the pol3-L612M* effects were milder, which would be in line with the lower rNMP incorporation rate of *POL3* compared to *POL2*^12^. Interestingly, we noticed a synthetic sick interaction between *rtt101* and *pol3-L612M* in the presence of high HU (Supplementary Fig. 5C, D). In conclusion, both mutator alleles boost genomic rNMP levels and enhance the rNMP-derived negative synthetic sick genetic interactions between RNase H2 dysfunction and *RTT101*.

In summary, we report that the negative genetic interaction between the deletion of Rtt101^Mms1-Mms22^ ubiquitin ligase subunits and RNase H2 defects is due to RER-deficiency and is exacerbated in rNMP accumulating (*pol2-M644G*, HU) condition. In RER-defective cells, rNMPs are likely hydrolyzed prior to, or during, S phase and require Rtt101 mediated HDR for repair upon encounter with the replisome. In line with the physical association with the replisome in S phase^19^, Rtt101 function is essential during S phase to counteract rNMP-derived cellular toxicity.

### Rtt101 may drive rNMP repair in S phase through histone H3 ubiquitylation

Using different genetic models (*S-RNH202* allele, RNase H2 deletion, alkaline conditions, *pol2-M644G* allele, *rnh201-RED* allele), we demonstrated that the Rtt101^Mms1-Mms22^ ubiquitin ligase complex is required to deal with Top1-independent rNMP-derived DNA damage in S phase. We speculate that we may have found the genetic requirements for a unique rNMP-derived lesion repair pathway that acts in S phase, complementing the RER and the Top1 pathways^14^. We set out to get a deeper molecular understanding by further probing the genetic interactions from the *S-RNH202* SGA genetic network (Fig. 1) and potentially identify substrates for Rtt101.

The Rtt101^Mms1-Mms22^ complex has previously been shown to ubiquitylate histone H3 on three lysine (K) residues (K121, K122, and K125)^20^. The modification does not lead to proteasomal degradation, but rather facilitates the deposition of newly synthesized histones during replication-coupled nucleosome assembly (RCNA). Other, non-replication related, substrates of Rtt101^Mms1-Mms22^ have been reported in yeast^29^, whereas multiple targets of Cul4 have been elucidated in human cells^30–35^.

**Fig. 4:**
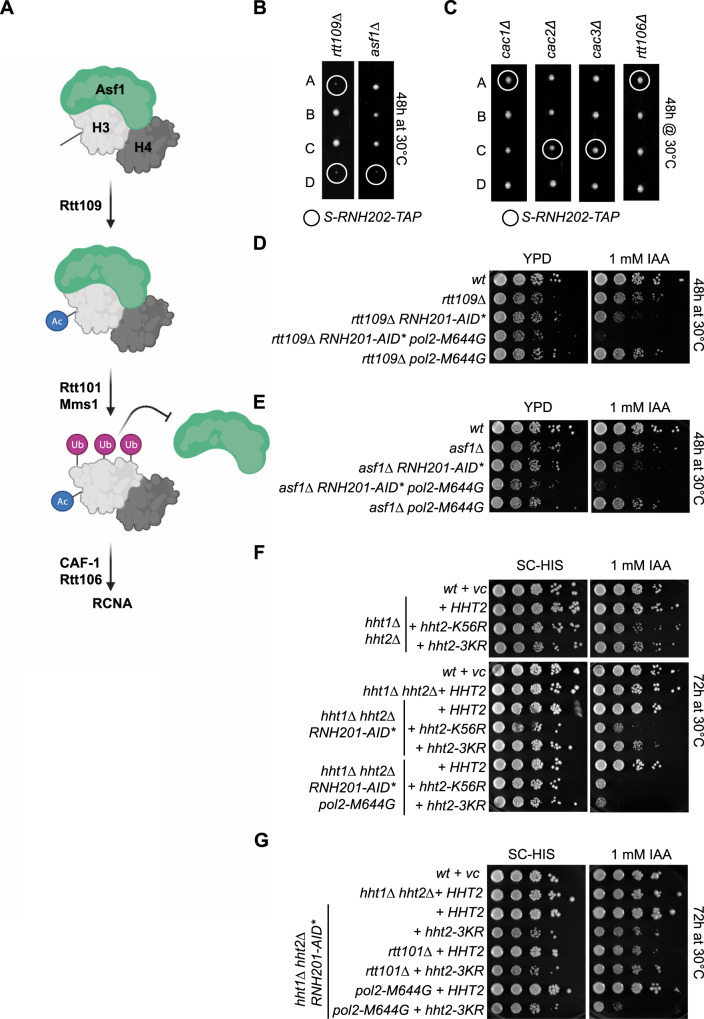
Rtt101 mediates the repair of rNMP-derived DNA damage in S phase through histone H3 ubiquitylation. **A** Scheme of the contribution of the histone chaperone Asf1, the histone acetyl transferase Rtt109 and the ubiquitin ligase Rtt101^Mms1^ to the replication-coupled nucleosome assembly (RCNA) pathway. Sequence of events (modified from ref. ^20^): Asf1 binds to *de novo* synthesized H3-H4 dimers and Rtt109 acetylates H3 followed by ubiquitylation of H3 through Rtt101 that lead to the release of Asf1 and facilitates DNA incorporation. Note that an acetylation deficient *H3-K56R* mutant is reminiscent of *RTT109* deletion and an ubiquitylation-deficient *H3-3KR* mutant is reminiscent of the *RTT101* deletion. Created with BioRender.com. **B** Manual tetrad dissection confirmed the synthetic lethal phenotype between the *S-RNH202* allele and the histone remodeler genes *RTT109* and *ASF1* (double mutant colonies in circles). **C** Representative tetrads from single CAF-1 complex deletion mutants (*cac1*Δ, *cac2*Δ, *cac3*Δ) in combination with *S-RNH202-TAP* to check contribution of the RCNA pathway. Note that these genes work redundantly and they are synthetic lethal with each other, hence we cannot exclude their contribution. **D** Spot assays with *RTT109* deletion mutants shows that loss of *RTT109* is toxic in RER-deficient strains with high rNMP load (*pol2-M644G*). **E** The same is true for loss of the histone chaperone *ASF1*. **F** Spot assays with histone H3 mutants deficient for Lysine-56 acetylation (“*H3-K56R*”) and Rtt101-dependent Lysine-121,122,125 triple ubiquitylation (“*H3-3KR*”)^20^. These plasmid-borne mutant versions of histone H3 replaced the two H3 coding genes (*HHT1* and *HHT2)* that were deleted from the genome (see yeast strain list for the respective complete genotypes). Histone H3 Lysine-56 acetylation became essential in RER-deficient cells (RNH201-AID* on IAA plates). The *H3-3KR* strain revealed mild sickness in RER-deficient cells but was inviable when rNMP levels increased with the *pol2-M644G* allele. **G** Strains from (**F**) were combined with *RTT101* deletion to confirm the epistasis between *RTT101*-deficiency and H3 ubiquitylation deficiency (compare lanes 4–6).

The histone chaperone Asf1 and the histone acetylase Rtt109, which acetylate lysine 56 of H3 (H3K56), act upstream of Rtt101^Mms1-Mms22^ in terms of nucleosome assembly^20^ (Fig. 4A). Similar to *RTT101*, the *ASF1* and *RTT109* genes were essential for cellular survival when rNMPs were nicked in the S phase (Fig. 4B). In the RCNA pathway, Rtt101 is responsible for the ubiquitylation of newly synthesized histone H3 on three lysine residues, which release the H3-H4 dimer from the histone chaperone Asf1 (Fig. 4A). As the downstream RCNA factors *CAC1/RLF2*, *CAC2*, *CAC3/MSI1* that form the CAF-1 complex and *RTT106* have redundant roles^36^, the single deletions do not affect *S-RNH202* colony growth (Fig. 4C). CAF-1 deletion combined with *RTT106* deletion is synthetic lethal, which is why we cannot rule out their contribution. We generated heterozygous diploid strains and derived the haploid double mutants to test the impact of Rtt109 and Asf1 loss in a RER-deficient condition using the RNH201-AID* degron and the *pol2-M644G* allele (Fig. 4D, E). Strikingly, triple mutants displayed lethality in the presence of auxin, reflecting the major role of RCNA factors, Asf1 and Rtt109, in the repair of rNMP-derived lesions (Fig. 4D, E).

To assess if the Rtt101-dependent H3 ubiquitylation has a direct role in rNMP-lesion repair, we combined the ubiquitylation-deficient *H3-3KR* mutant^20^ with an RER-deficient background using the RNH201-AID* degron (Fig. 4F). Rtt109-mediated H3-K56 acetylation occurs upstream of Rtt101-dependent H3 ubiquitylation^20^. The *H3-K56R* acetylation-deficient strain was synthetic sick with loss of RNase H2 and inviable when rNMPs accumulate in the RNH201-AID* pol2-M644G strain background (Fig. 4F). Interestingly, the *H3-3KR* ubiquitylation-deficient mutant could support growth upon loss of RNase H2 better than the H3-K56R mutant, however, Rtt101-dependent H3-ubiquitylation became essential when rNMP load increased in the RNH201-AID* pol2-M644G strain (Fig. 4F). To show that Rtt101 and the Rtt101-dependent H3 ubiquitylation behave in an epistatic manner, we deleted *RTT101* in the *H3-3KR* mutant strains. Indeed, deleting *RTT101*, or impairing H3 ubiquitylation (*H3-3KR*), or the combination of both impaired cell viability to the same degree in RER-deficient strains (Fig. 4G). This suggests that histone H3 is a key target of Rtt101, and we conclude that Rtt101^Mms1-Mms22^ dependent histone H3 ubiquitylation at lysines-121, -122, and -125 is critical for the repair of rNMP-derived DNA damage. However, it also suggests that other functions of the RCNA pathway may be important for rNMP repair in RER defective strains. Interestingly, Asf1 has a role in the regulation of Rad53 checkpoint control^37^. It will be important to unravel the unknown connections that still exist between Rtt101, DNA repair, checkpoint recovery, and chromatin (RCNA).

## Discussion

Eukaryotic cells repair genomic rNMPs by RNase H2-initiated ribonucleotide excision repair (RER). The loss of RNase H2 function leads to the accumulation of genomic rNMPs, which then become, to an extent, substrates for error-prone repair by Top1 (reviewed in^14^). Recently, it has been demonstrated that some human cancers harbour RNase H2 mutations, resulting in rNMP accumulation and Top1-mediated genome instability^6^. These cancers are considered “druggable” as Top1 lesions recruit PARP to sites of damage and hence become susceptible to PARP inhibitors^6^. Elucidating alternative rNMP repair pathways may yield additional factors and pathways that could potentially be targeted in RER defective human cancer cells. Importantly, it has been shown that RER-defective budding yeast have a nearly identical mutagenic signature profile as RER defective cancer cells^11^, hence making yeast a highly relevant model for the study of rNMP repair.

The loss of *RAD52* becomes essential in RER-defective yeast cells and *TOP1* deletion can only partially rescue the loss of fitness, indicating that there might be additional sources for rNMP-mediated genome instability apart from Top1^8,38^. Genomic rNMPs are prone to hydrolysis and nick formation and it was shown recently that the CMG helicase will eventually run off the DNA, if the leading strand template is nicked upstream of the replication fork^39^. Hence, we hypothesized that the HDR machinery was repairing rNMP-induced lesions (seDSB) that occur when a nicked rNMP encounters replication^8^. In support of this idea, increased rNMP-nicking in RER-deficient cells rendered cells dependent on HDR, independent of Top1^8^.

**Fig. 5:**
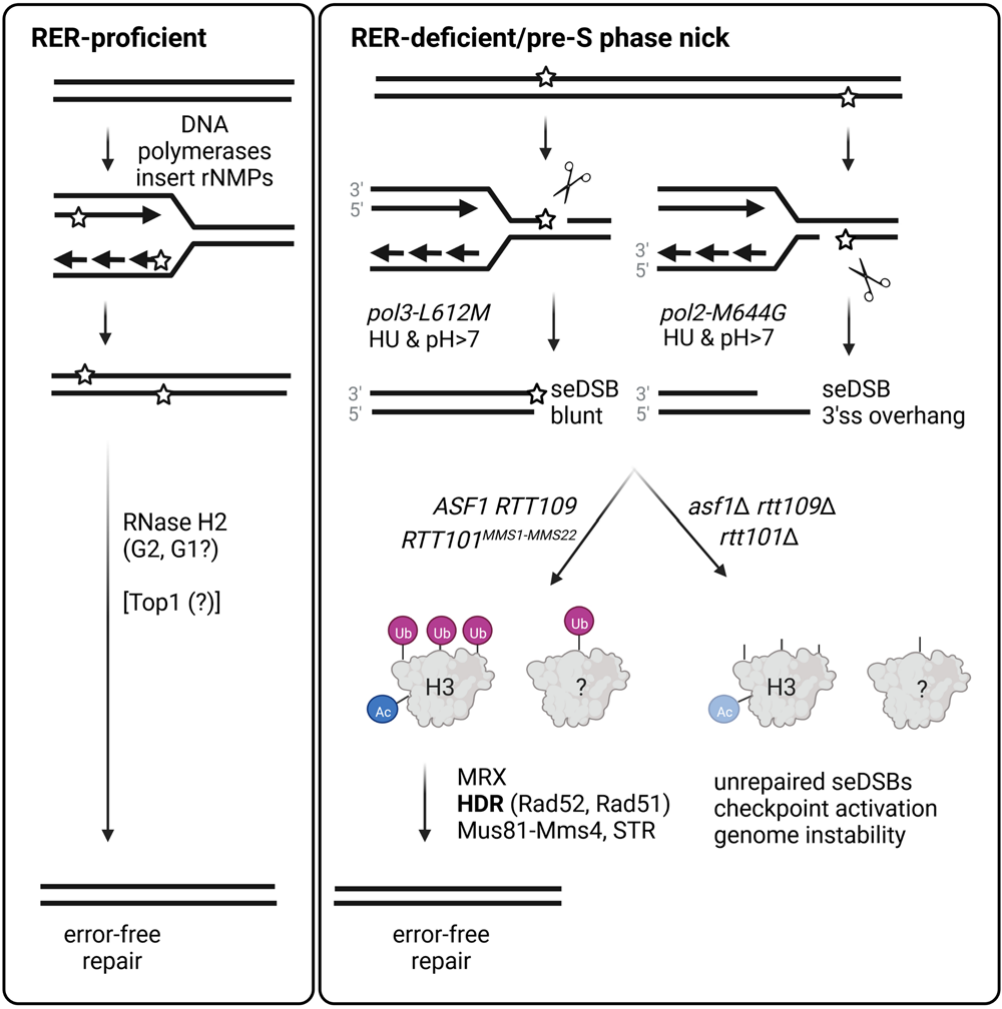
Top1-independent NLR pathway is essential when rNMPs cause pre-S phase nicks that result in seDSB. DNA polymerases transiently incorporate single rNMPs into the genome during replication and repair. The RER pathway removes genomic rNMPs immediately in the subsequent G2 phase. In RER-deficient, or RER-dysfunctional cells the Top1-mediated backup pathway deals with rNMP-removal. However, if high amounts of persistent genomic rNMPs accumulate in RER-deficient, or RER-dysfunctional cells, the likelihood increases that hydrolysis-prone rNMPs form ssDNA nicks. When the replication fork encounters such an rNMP-derived nick, a toxic seDSB is formed. End topology of the seDSB may differ dependent on the location of the nick^39^. To repair the rNMP-derived seDSB lesions, functional RCNA is required. The histone remodelers Asf1 and Rtt109 act upstream of Rtt101^Mms1-Mms22^, presumably accompanied by the resection of the seDSB by MRX (Mre11-Rad50-Xrs2), followed by HDR (Rad52, Rad51) and resolution of the HDR intermediates (Mus81-Mms4, Sgs1-Top3-Rmi1) to result in the error-free repair of the seDSB. In *RTT101*-deficient cells with high rNMP load, histone H3 does not become ubiquitylated and downstream error-free HDR repair of the seDSB is compromised causing genomic instability likely by alternative, error-prone repair attempts. Abbreviations: NLR rNMP-derived nick lesion repair, rNMP single ribonuclesoide monophates, RER ribonucleotide excision repair, seDSB single-ended double strand break, ssDNA single stranded DNA, RCNA replication-coupled nucleosome assembly, HDR homology-directed repair. Created with BioRender.com.

Here, we employed the *S-RNH202* allele to induce seDSBs at rNMPs to look for mutants with reduced fitness similar to *rad52*Δ, in a genome-wide screening approach (Fig. 1). As a result, we elucidated a genetic network for rNMP-derived nick lesion repair (NLR) (Fig. 5). NLR includes the Rtt101 ubiquitin ligase, the Rad52-based HDR machinery, the MRX (Mre11-Rad50-Xrs2) complex, and the Rtt109/Asf1 replication-coupled nucleosome assembly (RCNA) pathway (Fig. 1). In addition, we found the STR (Sgs1-Top3-Rmi1) and Mus81-Mms4 complexes, which likely provide resolution of the multiple recombination intermediates formed during HDR^40^ (Fig. 1). We showed that the exclusive nicking of rNMPs in S phase is particularly toxic in *rtt101*Δ cells (Fig. 2A). Indeed, we could demonstrate that loss of *RAD51* and *RTT101* are epistatic in terms of rNMP repair (Fig. 2G). Furthermore, we confirmed that loss of *RTT101* was sufficient to kill RER-deficient cells in a Top1-independent manner (Fig. 3E). We were also able to conclude that Rtt101 function is required in S phase (Fig. 3H), which is in alignment with its replisome association^19^. Moreover, the mutated allele of histone H3 that can no longer be ubiquitylated by Rtt101 (H3-3KR) also renders cells highly sensitive to high levels of rNMPs (Fig. 4F–G). Although Rtt101, HDR and H3 ubiquitylation are all working together in a genetic pathway it remains unclear as to how Rtt101 promotes HDR.

One possibility would be that rNMPs are more susceptible to induce nicks because the chromatin structure of *rtt101*Δ cells is altered due to the RCNA defects. This would be consistent with decreased nucleosome deposition and a more open chromatin state. In agreement, it has been reported that telomeric heterochromatin is lost in *rtt101*Δ and *mms1*Δ mutants^21^. It will be important determine if hydrolyzed rNMPs are more frequent in more accessible chromatin environments and if such environments actually increase in the absence of the Rtt101 complex. Alternatively, it could be that Rtt101-mediated H3 modifications are important for the HDR reaction itself. This hypothesis is supported by the fact that deletion of the fork protection protein and damage checkpoint mediator *MRC1* can rescue the sensitivity of *rtt101*Δ cells to genotoxic agents^19^ as well as to accumulation of rNMPs (Fig. 2A). Indeed, Mrc1 can differentially regulate resection and HDR at DSBs^41^ and it was recently demonstrated that this involves changes in chromatin compaction^42^. Further support that the repair of rNMP-derived lesions is coupled to alterations of chromatin was shown in a recent study in human cells^43^. Specifically, they looked at seDSB damage caused by the replisome running into Top1-DNA adducts after CPT treatment. In line with our yeast genetic network, HDR factors, MRN, RAD51, and MMS22L-TONSL were found to be associated with the broken forks in human cells^43^. Interestingly, these broken and stalled replication forks presented a distinct chromatin environment with a defect in histone deposition^43^.

In addition, sister chromatin cohesion is important at seDSBs to ensure that repair occurs primarily from the sister chromatid and not a homologous chromosome. Rtt101, Mms1, and Mms22 promote sister chromatid cohesion through their replisome association^44^. Interestingly, the cohesion-like Smc5/6 complex becomes essential in the absence of RER (^45^) and may also be intertwined with the Rtt101, Rtt109, HDR-mediated repair of rNMPs. In fission yeast, the mega-nuclease complex MRN (Mre11-Rad50-Nbs1) is critical to control sister chromatid cohesion at replication-associated seDSBs to allow HDR repair and prevent Ku-mediated DSB repair^46^. In accordance, all subunits of the *Saccharomyces cerevisiae* MRX complex seem to be essential for NLR (Supplementary Fig. 1I). The restriction to the 4790 non-essential genes, covering 75% of the total 6275*S. cerevisiae* ORFs, is one important limitation of this study. However, the comparative analysis to a published dataset including essential genes allowed us to pinpoint the *NSE4* gene, a subunit of the Smc5/6 complex as a factor relevant for NLR (Supplementary Fig. 2E). Since cohesin and cohesin-like factor are essential, they were not revealed in our screen and will have to be systematically tested using conditional alleles.

Our current working model integrates the NLR network into a repair pathway occurring after a nicked DNA template would lead to the formation of a seDSB during replication (Fig. 5). According to recent in vitro work using *Xenopus laevis* extracts, the fate of a replication fork encountering a nicked DNA template is the formation of a seDSBs both with a leading strand template nick and a lagging strand template nick^39^. Through the use of polymerase mutants that incorporated rNMPs in a strand specific manner, we determined that the Rtt101 E3 ligase became essential at rNMPs in a strand independent manner. It will be important to determine whether the Rtt101 E3 human equivalent, Cullin-Ring-Ligase 4 (CRL4), also contributes to rNMP repair in RNase H2 defective cells, as this may represent alternative therapeutic opportunities, in addition to PARP inhibitors in RER defective cancer cells. It is feasible to put this to the test in the future as the CRL neddylation inhibitor MLN4924 was extensively studied and went into clinical trials for cancer intervention^47,48^. In this respect, it is interesting that the cullin subunit of CRL4 (CUL4A) is overexpressed in many human cancers^49^. The cancer-specific overexpression is a result of the genomic locus in human cells that undergoes amplification in cancers^50^. Hence, it is possible that this overexpression promotes CRL4-dependent DNA repair also in the context of other human deficiencies (e.g., RER). We speculate that the role for NLR could be greater than expected as the inside of a cancer cell is slightly alkaline (pH > 7), and could therefore promote rNMP-mediated hydrolysis of the DNA backbone. The intracellular alkalization of cancer cells seems connected to the initial oncogenic transformation and the progression of the tumour^51,52^. Translational studies will show if RER-defective human cancer cells with alkaline intracellular environment may even favour NLR due to augmented spontaneous rNMP hydrolysis. The NLR genes comprise DNA repair complexes that participate in a variety of well-characterized DNA repair pathways. However, in this combination, they have not been described elsewhere to our knowledge. Especially the connection of DNA repair (recombination, resection, unwinding) with chromatin remodelling (RCNA) is interesting and requires further study and careful separation of the multiple roles of e.g., MRX and STR complexes. Hence, we are favoring the idea that NLR could be relevant for the repair of all kinds of replisome-nick encounter-derived lesions in cycling cells with the common feature of a toxic seDSB as the damage site.

## Methods

### Yeast strains and plasmids

*Saccharomyces cerevisiae* strains used in this study derive of the standard S288C (*MATa his3*Δ*1 leu2*Δ*0 ura3*Δ*0 met15*Δ*0*) strain and are listed in Supplementary Data 2. Strains were grown under standard conditions in YPD (1% [w/v] yeast extract, 2% [w/v] peptone supplemented with 2% glucose) or in SC (0.2% [w/v] Synthetic Complete medium without specific amino acids, 1% [w/v] yeast nitrogen base supplemented with 2% glucose) at 30 °C if not indicated otherwise. Yeast transformations with plasmid or PCR products were performed with the standard lithium acetate polyethylene glycol (PEG) method^53^. Gene deletions, tagging, and generation of specific mutations were performed using standard PCR-based recombination methods and confirmation by sequencing. Plasmids and oligonucleotides are listed in Supplementary Data 3.

### Yeast tetrad dissection

For analysis of the meiotic product, we crossed a *MATa* with a *MATalpha* haploid strain, selected for diploids based on auxotrophy or antibiotic resistance, and patched the diploid strain on rich pre-Sporulation plates (YP agar with 6% [w/v] glucose]. Then we froze part of the patch and transferred part of the patch into Sporulation medium (1% potassium acetate, 0.005% zinc acetate buffer) and incubated the cultures with shaking at 23 °C. After a few days, the sporulation cultures were treated in a ratio of 1:1 [v/v] with Lyticase (L4020 Sigma Aldrich, 2.5 mg/ml, 200 units/µl, in 1 M D-Sorbitol) to digest the ascus. After 15–20 min at room temperature, the culture was applied to an agar plate and tetrads were dissected using a Singer micromanipulator. Colonies of haploid spores grew at 30 °C for three days. Images were taken at 48 and 72 h with the ChemiDoc™ Touch Imaging System (Bio-Rad). After three days, the spores were replica plated, genotypes were scored and strains were frozen in 15% glycerol-containing cryopreserved stocks at −80 °C. Strains are listed in Supplementary Data 2.

### Flow cytometry analysis for DNA content

Cells were fixed in 70% ethanol overnight and then treated with 0.25 mg/ml DNase- and Protease-free RNase A (ThermoFisher Scientific, 10753721) at 37 °C for 2 h and Proteinase K (Biofroxx, 1151ML010) at 50 °C for 2 h in 50 mM Tris-HCl pH7.5 buffer. The cell suspension was sonified using a Branson sonifier 450 for 5 s with output control 1 and duty cycle constant. Then, cells were stained with a final concentration of 2.4 µM SYTOX Green nucleic acid stain (ThermoFisher Scientific, 1076273). Measurement was performed on the BD LSRFortessa flow cytometer (BD Biosciences) using the BD FACSDiva software (v9.0.1). With low flow rate, 20,000 events were recorded. Analysis was performed with FlowJo (v10.8.0) using the following gating strategy: From the main population in FSC-A vs. SSC-A, doublets were excluded in the SYTOX Green A vs. W channel, and DNA content was assessed in the histogram of the SYTOX Green-A channel (Ex 488 nm, 530/30BP).

### Flow cytometry analysis for cell viability

In contrast to measurements of the DNA content using ethanol-fixed dead cells, we collected exponentially growing cells and, without fixation, stained them with SYTOX Green to discriminate between living cells (do not take up SYTOX Green) and dead cells (will incorporate SYTOX Green).

Cells were collected and the cell pellet was washed with 50 mM Tris pH 7.5 and resuspended in 1 ml 50 mM Tris pH 7.5 containing 0.5 μM SYTOX Green. Measurement and analysis were the same as for the DNA content analysis except for doublet exclusion, which was done in the SSC-A vs. W channel. As a control sample for dead cells, controls were incubated at 95 °C for 15 min and subjected to the described protocol.

### Protein extraction, SDS-PAGE, and western blot

Proteins were extracted from 2 OD_600_ units of yeast cells as described in ref. ^54^. Protein extracts were loaded on precast Mini-PROTEAN TGX precast gels (Bio-Rad). Proteins were blotted on a nitrocellulose membrane with the Trans-Blot Turbo Transfer System (Bio-Rad). The membrane was fixed with Ponceau S solution (P7170, Sigma Aldrich) and blocked for 1 h with 5% skim milk in 1xPBS containing 0.001% Tween-20 (PBS-T). The primary antibodies were incubated overnight in 5% skim milk in PBS-T. Peroxidase coupled secondary antibodies were incubated for 1 h at room temperature. Antibodies are listed in Supplementary Data 4. Antibodies were used in the following dilutions: anti-Rad53 (Abcam, Cat#ab166859, dilution 1:1000), Rabbit Peroxidase Anti-Peroxidase soluble complex (Sigma-Aldrich, Cat#P1291, dilution 1:2000), Mouse monoclonal anti-Phosphoglycerate Kinase 1 (22C5D8) (Invitrogen, Cat#459250, dilution 1:10,000), Mouse anti-Myc-Tag (9811) (Cell Signalling, Cat#2276S, dilution 1:1000), Goat Immun-Star anti-mouse (GAM)-HRP conjugate (Bio-Rad, Cat#170-5047, dilution 1:3000), Goat Immun-Star anti-rabbit (GAR)-HRP conjugate (Bio-Rad, Cat#170-5046, dilution 1:3000). The western blots were developed using the Super Signal West Pico Chemiluminescent Substrate (Thermo Scientific) and the ChemiDoc™ Touch Imaging System (Bio-Rad).

### Construction of strains with auxin-inducible degron

Strains carrying the auxin-inducible degron (AID*) for RNase H2 (catalytic subunit Rnh201) were created as described before^27^. Plasmids and oligonucleotides are listed in Supplementary Data 3.

### Construction of the cell cycle restricted RNase H2 alleles

The *S-RNH202-TAP-HIS3* and *G2-RNH202-TAP-HIS3* alleles were described previously^8^.

The *G1-RNH202-TAP-HIS3* allele was created by amplifying the “G1 cassette” using the oligos oNA21 and oNA22 with the template pBL603 (containing the *SIC1* promoter, the first 315 bp of the *SIC1* gene and the NAT resistance cassette)^55^ by PCR^56^. Transformed colonies were grown under selective pressure, and sequence verified by sequencing with the respective oligonucleotide pairs. The cell cycle specific expression was confirmed by western blot. Plasmids and oligonucleotides are listed in Supplementary Data 3.

### α-factor arrest and release

For cell cycle analysis, cells were synchronized in G1 phase by addition of 4 µg/mL α-factor (Zymo research, mating hormone peptide) for 2 h. Cells were then spun and washed three times with water, released into fresh YPD medium and further grown at 25 °C in a water bath. Protein and Flow cytometry samples were collected at indicated time points.

### Canavanine mutagenesis assay

The *CAN1* fluctuation analysis was performed as described in^57^. Relevant genotypes for the *Can*^*R*^ mutation assay were streaked out 48 h prior inoculation to conserve population doublings within replicates. At least 14 independent single colonies from each genotype were entirely excised from the agar plate using a sterile scalpel to inoculate a 10 ml of YPD medium. The cultures were incubated at 30 °C, 250 rpm for 16 h. After measuring the optical density of the cultures they were harvested by centrifugation. Then, each culture was resuspended in 1 ml sterile water. Exactly 1 ml of each resuspension was transferred to a new tube. From this, a 10-fold dilution series up to a dilution factor of 10^6^ was performed in a 96-well plate. Finally, 100 µl of all strains from the 10^−6^ dilution were plated on a YPD plate and distributed with exactly four glass beads per plate. All strains were plated on SC-ARG plates supplemented with 60 µg/mL canavanine with the indicated dilution factor. The plates were incubated for 72 h at 30 °C before the outgrown colonies were manually counted. The medium for the *Can*^*R*^ mutation assay was mixed, autoclaved and poured each day before plating to maintain constant conditions between replicates.

For evaluation, the number (#) of mutant cells per culture, representing *r*, was calculated:

1 $$r={culturevol}.\times \left({conc}.\;{factor}\times \frac{{{{{{\rm{\#}}}}}}{mutant}\,{colonies}}{{plated}\,{vol}.}\times {dilution}\,{factor}\left({plated}\right)\right)$$

The following correction was used to account for the progenies of each individual *CAN1* mutation event per cell. With *M* being a scaled value that represents the number of cells that have actually undergone a mutation event (from which the counted progenies originated):

2 $$r=M(1.24+{{{{{\rm{ln}}}}}}M)$$

The final mutation rate was calculated dividing *M* by the total number of cells present in the initial culture:

3 $${mutation}\,{rate}=\frac{M}{{{{{{\rm{\#}}}}}}{cells}\,{in}\,{the}\,{culture}}$$

The data was plotted as the Median with 95% Confidence interval using the GraphPad PRISM8 software.

### Plating assay

Exponential cultures at 30 °C were synchronized with α-factor for 1 h and then split to start the degradation of Rnh201-AID*-9Myc with 1 mM IAA (auxin) in 50% of the samples during the residual 1 h of synchronization. Half of the culture remained arrested in G1 phase and the other half was released into S phase, by washing out α-factor, in the presence or absence of 1 mM auxin for 30 min. Of each culture and condition, a suitable dilution was empirically determined that yielded in 100–200 colonies per YPD agar plate after outgrowth. The plates were incubated for 2 days at 30 °C. Colonies of 7 replicates were manually counted and adjusted for differences in optical density (OD_600_) before dilution. Statistical analysis and plot generation was performed using Prism8 (GraphPad Software).

### Yeast spot assay

Single colony derived yeast cells were incubated overnight at the appropriate temperature in liquid medium. Cells were diluted to 0.5 OD_600_ and spotted in ten-fold serial dilutions onto YPD plates, SC plates, or plates containing the indicated amount of genotoxic drugs, i.e., methylmethanesulfonate (MMS), camptothecin (CPT) or hydroxyurea (HU) (all drugs: Sigma-Aldrich). The agar plates were incubated at the indicated temperatures and time and imaged using the ChemiDoc™ Touch Imaging System (Bio-Rad).

Standard YPD agar has pH 5.5. To make YPD agar plates with alkaline pH, we titrated melted YPD agar with 10 N NaOH until pH 8.0 was reached. The *wsc1::KAN* knockout strain was used as a positive control for the alkaline agar plates^28^.

### Liquid yeast growth curves

Yeast cells were diluted to 0.05 OD_600_ in 100 µl of liquid growth medium, or medium containing drugs at indicated concentrations and transferred to a 96-well plate (Falcon®). Hourly OD_600_ measurements were performed at 30 °C for 20 h by using a Tecan Spark® microplate reader. Growth curves were evaluated in Microsoft Excel. The linear range of each curve was plotted to find the linear fit with the formula y=Ye^Bx. The PD was determined using the log naturalis ln=0.6931 and the formula PD = ln/B*60 min. The PD plots were generated in GraphPad PRISM8.

### Alkaline gel electrophoresis

Analysis of alkaline-labile sites in genomic DNA was performed as reported earlier^1^. Briefly, overnight cultures were diluted for exponential growth in 50 ml medium and harvested at the optical density of OD_600_ = 0.6. Genomic DNA was isolated with the Qiagen Gentra Puregene Yeast/Bact Kit (Qiagen, 158567). The gDNA concentration was determined by an intercalating fluorescent dye supplied via the Qubit dsDNA broad-range kit (Invitrogen, Q32854). In a final volume of 40 µl, 10 µg of extracted gDNA were treated, shielded from light, for 2 h at 55 °C with 0.3 M KOH or 0.3 M KCl. KOH treated DNA samples were mixed with 8 µl 6× alkaline loading buffer (300 mM KOH, 6 mM EDTA, 18% (w/v) Ficoll Type 400, 0.15% (w/v) bromocresol green, 0.25% (w/v) xylene cyanol) before loading on a 1% alkaline agarose gel; the alkaline gel was casted using the 1× dilution of the 10× alkaline running buffer (0.5 mM NaOH, 10 mM EDTA). The alkaline gel was run in 1× alkaline running buffer. The KCl treated gDNA samples were loaded on a 1% TBE agarose gel with standard loading dye. Both gels were run at room temperature for 5 min at 65 V before reducing the voltage to 26 V for 18 h. The alkaline gel was neutralized in two washes of 250 ml (700 mM Tris-HCl pH 8.0, 1.5 M NaCl) for 45 min each with gentle shaking. Both gels were stained in a 1:10,000 SyBr Gold solution (ThermoFisher, S11494) (diluted in 1×TBE for the neutral gel; diluted in water for the alkaline gel) for 2 h with gentle shaking before image acquisition.

### Construction of cell cycle regulated *RNH202* allele in the SGA query strain background

The *G1-RNH202-TAP (this study)*, *S-*, and *G2-RNH202-TAP* alleles^8^ were crossed to the haploid background strain (Y8205, Source C. Boone) for the SGA query strain construction. Selection of diploids, sporulation and tetrad analysis generated the four query strains used in SGA analysis. Cell cycle restricted protein expression of Rnh202 was confirmed by arrest and release experiment and western blot analysis. The selectable markers for SGA analysis were verified by PCR (oMT86/oMT91 for *can1Δ::STE2pr-Sp_his5*, oMT89/oMT90 for *lyp1Δ::STE3pr-LEU2*) and replica-plating on YPD + (50 μg/ml canavanine, 50 μg/ml thialysine). The yeast strains are listed in Supplementary Data 2.

### Synthetic genetic array (SGA) screen procedure and data evaluation

The *G1-*, *S-*, and *G2-RNH202-TAP* query strains and a wild type *RNH202* control query were crossed with the haploid genome-wide library of yeast gene deletion mutants, the YKO^58^. Crosses were performed in 1536-colony format, with the four queries combined on each screen plate, with four technical replicates of each cross, arranged next to each other. To minimize spatial effects, four outer rows and columns contained dummy strains. Mating, sporulation and selection of haploids carrying both a query allele (cell cycle regulated *RNH202* alleles or wild type control) and a gene deletion were performed by sequential pinning of yeast colonies on appropriate selective media using a RoToR pinning robot (Singer Instruments) as described^59^. Plates with the final colony arrays were imaged after 24 h with the Singer PhenoBooth colony imager. Data analysis was performed in R (R Core Team (2021). R: A language and environment for statistical computing. http://www.R-project.org/) as detailed in https://github.com/Khmelinskii-Lab/nick_lesion_repair_SGA_screen. Briefly, photographs of colony arrays were segmented using the gitter package^60^ to determine colony size. Measurements of empty positions and four outer rows and columns were assigned NA values. Colony size measurements on each plate were corrected for spatial effects using the SGA tools package^60^ and normalized to the median on each plate. Genetic interactions in double mutants were identified under the assumption of multiplicative combination of effects of single mutants in the absence of genetic interactions^59^. For that, normalized colony size measurements were divided by the median per query to obtain normalized double mutant fitness. For each mutant in the YKO collection, differences between crosses with a cell cycle and the wild type queries were assessed with a t-test, excluding replicates contributing more than 90% of variance. The *p*-values were adjusted for multiple testing using the Benjamini-Hochberg method. Finally, replicates were summarized by their mean, excluding replicates contributing more than 90% of variance (Supplementary Data 5). Negative genetic interactions were verified through manual generation of haploid double mutants, by crossing single colonies from the YKO haploid collection to the *S-RNH202* allele, selection for diploids, sporulation and tetrad analysis. False positives and linked genes have been excluded in the final analysis (greyed out).

### Materials

Materials such as antibodies, enzymes, and chemicals are listed in Supplementary Data 4.

### Reporting summary

Further information on research design is available in the Nature Portfolio Reporting Summary linked to this article.

## Supplementary information

Supplementary Information

Description of Additional Supplementary Files

Supplementary Data 1

Supplementary Data 2

Supplementary Data 3

Supplementary Data 4

Supplementary Data 5

Reporting Summary

## Data Availability

Underlying numerical data for all graphs and original images for western blots are provided as a Source Data file with this paper. The data supporting the findings of this study are available from the corresponding author upon request. Source data are provided with this paper.

## Source data

Source Data

## Code availability

Codes for the SGA data analyses in this study are available in github (https://github.com/Khmelinskii-Lab/nick_lesion_repair_SGA_screen).
