# Supplementary material for: Genetic requirements for repair of lesions caused by single genomic ribonucleotides in S phase
Source: Nat Commun. 2023 Mar 3;14:1227. doi: 10.1038/s41467-023-36866-6 (PMC9984532; doi:10.1038/s41467-023-36866-6)
Supplement: Supplementary file 1 — Supplementary Information [file 41467_2023_36866_MOESM1_ESM.pdf]

Genetic requirements for repair of lesions caused by single genomic ribonucleotides in S phase

Natalie Schindler[1,*,#], Matthias Tonn[1,*], Vanessa Kellner[2,3], Jia Jun Fung[2], Arianna Lockhart[2], Olga Vydzhak[1], Thomas Juretschke[2], Stefanie Möckel[2], Petra Beli[2], Anton Khmelinskii[2], and Brian Luke[1,2,#]

[1]Johannes Gutenberg University Mainz, Institute for Developmental Neurology (IDN), Biozentrum 1, Hanns-Dieter-Hüsch-Weg 15, 55128 Mainz, Germany

[2]Institute of Molecular Biology (IMB), Ackermannweg 4, 55128 Mainz, Germany

[3]present address: Department of Biology, New York University, New York, NY, USA

[#]Correspondence: b.luke@imb-mainz.de; natalie.schindler@uni-mainz.de

*these authors contributed equally to the manuscript

Supporting information

Supplementary Figures 1-6

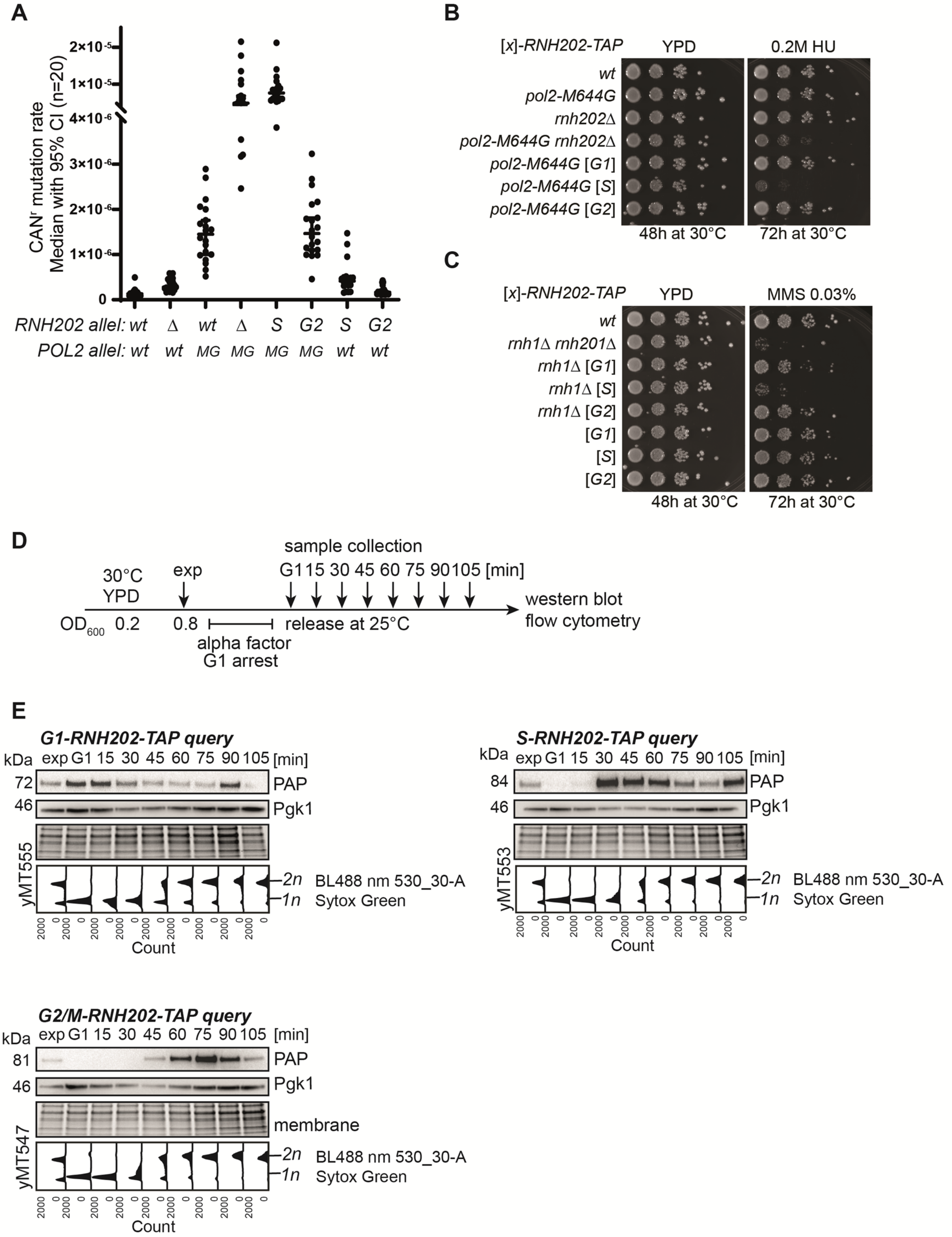
A
CAN^r mutation rate
Median with 95% CI (n=20)
2×10^-5
1×10^-5
4×10^-6
3×10^-6
2×10^-6
1×10^-6
0
RNH202 allel: wt Δ wt Δ S G2 S G2
POL2 allel: wt wt MG MG MG MG wt wt
B
[x]-RNH202-TAP
YPD
0.2M HU
wt
pol2-M644G
rnh202Δ
pol2-M644G rnh202Δ
pol2-M644G [G1]
pol2-M644G [S]
pol2-M644G [G2]
48h at 30°C
72h at 30°C
C
[x]-RNH202-TAP
YPD
MMS 0.03%
wt
rnh1Δ rnh201Δ
rnh1Δ [G1]
rnh1Δ [S]
rnh1Δ [G2]
[G1]
[S]
[G2]
48h at 30°C
72h at 30°C
D
sample collection
30°C
YPD
exp
G1 15 30 45 60 75 90 105 [min]
western blot
flow cytometry
OD600 0.2
0.8
alpha factor
G1 arrest
release at 25°C
E
G1-RNH202-TAP query
kDa exp G1 15 30 45 60 75 90 105 [min]
72 PAP
46 Pgk1
yMT555
2n BL488 nm 530_30-A
1n Sytox Green
Count
S-RNH202-TAP query
kDa exp G1 15 30 45 60 75 90 105 [min]
84 PAP
46 Pgk1
yMT553
2n BL488 nm 530_30-A
1n Sytox Green
Count
G2/M-RNH202-TAP query
kDa exp G1 15 30 45 60 75 90 105 [min]
81 PAP
46 Pgk1
membrane
yMT547
2n BL488 nm 530_30-A
1n Sytox Green
Count

F

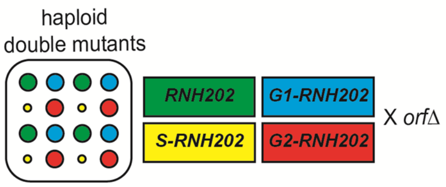
haploid
double mutants
RNH202
G1-RNH202
S-RNH202
G2-RNH202
X orfΔ

G

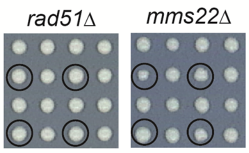
rad51Δ
mms22Δ

H

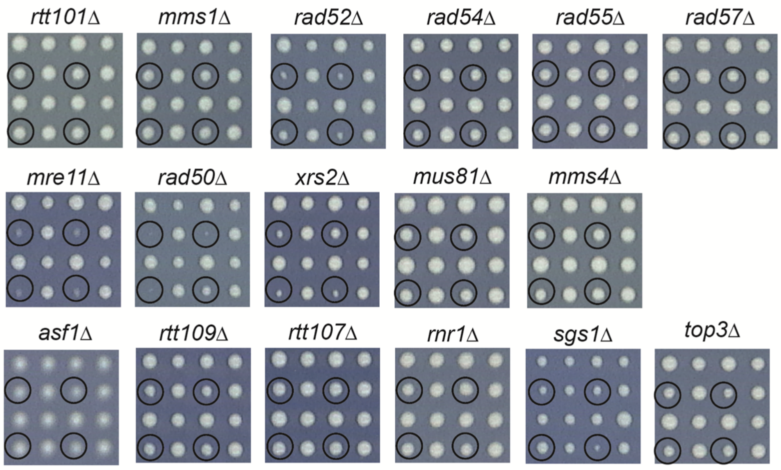
rtt101Δ
mms1Δ
rad52Δ
rad54Δ
rad55Δ
rad57Δ
mre11Δ
rad50Δ
xrs2Δ
mus81Δ
mms4Δ
asf1Δ
rtt109Δ
rtt107Δ
rnr1Δ
sgs1Δ
top3Δ

I

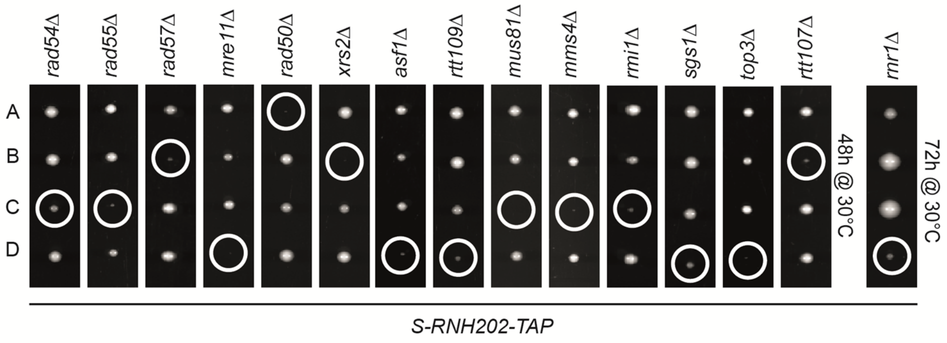
rad54Δ
rad55Δ
rad57Δ
mre11Δ
rad50Δ
xrs2Δ
asf1Δ
rtt109Δ
mus81Δ
mms4Δ
rmi1Δ
sgs1Δ
top3Δ
rtt107Δ
rnr1Δ
A
B
C
D
48h @ 30°C
72h @ 30°C
S-RNH202-TAP

J

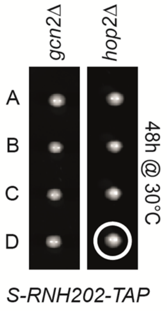
gcn2Δ
hop2Δ
A
B
C
D
48h @ 30°C
S-RNH202-TAP

K

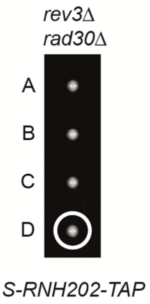
rev3Δ
rad30Δ
A
B
C
D
S-RNH202-TAP

Supplementary Figure 1

(A-C) Features of the *S-RNH202* allele. (A) Fluctuation assay for Canavanine-resistance mutagenesis showed that the *S-RNH202-TAP* allele has the same mutagenesis load as the RER-deficient *rnh202*Δ strain in presence of *pol2-M644G*. (**B)** Spot assay to compare the RNase H2 cell cycle alleles and deletion in the presence of high rNMP load (*pol2-M644G*). HU (hydroxy urea) reduced viability of the RER-deficient *pol2-M644G rnh202*Δ strain, while the *pol2-M644G S-RNH202-TAP* strain was inviable. Note that *G1-RNH202-TAP* and *G2-RNH202-TAP* alleles support full viability. **(C)** Serial dilution of *RNH202-TAP* cell cycle alleles in combination with *RNH1* knockout to assess their R-loop removal activity. MMS (methyl methanesulfonate) is a drug that accumulates R-loop levels in cells. **(D-K) Query strain characterization and representative SGA raw data. (D-E)** Western blot confirmed the cell cycle specific expression of the query strains after release from α-factor synchronization. DNA profiles served as control for the cell cycle phase. Note that these strains have the mating type *MATa* (susceptible to α-factor arrest); for the SGA screen in Figure 1, the corresponding *MATalpha* strains from the same dissection were used. **(F)** Schematic overview of genotypes in a SGA panel. Scheme was created using Adobe Illustrator. Every set was pinned in n=4 replicates. **(G)** Selected examples from the original SGA screen that scored below threshold, but were manually verified due to their context with the candidate genes in Figure 1G. *S-RNH202 orf*Δ double mutants are circled. **(H)** Selected examples from the original SGA screen that scored above threshold and were manually verified (see panel I). *S-RNH202 orf*Δ double mutants are circled. **(I)** Manual crosses of identified genes confirming the negative genetic interaction with the *S-RNH202-TAP* allele. The yeast knockout collection strain identified as *top3*Δ exhibits a negative genetic interaction with *S-RNH202-TAP*, but lacks the barcoded deletion cassette at the *TOP3* locus. **(J)** Representative manual crosses of identified genes without negative genetic interaction with the *S-RNH202-TAP* allele. Dissection of genes e.g. *GCN2/YDR283C* linked to the query strain *RNH202/YDR279W* resulted in four single mutants. **(K)** Compromised translesion synthesis had no effect on the *S-RNH202-TAP* fitness (Pol eta and Pol zeta double deletion highlighted).

Source data are provided as a Source Data file

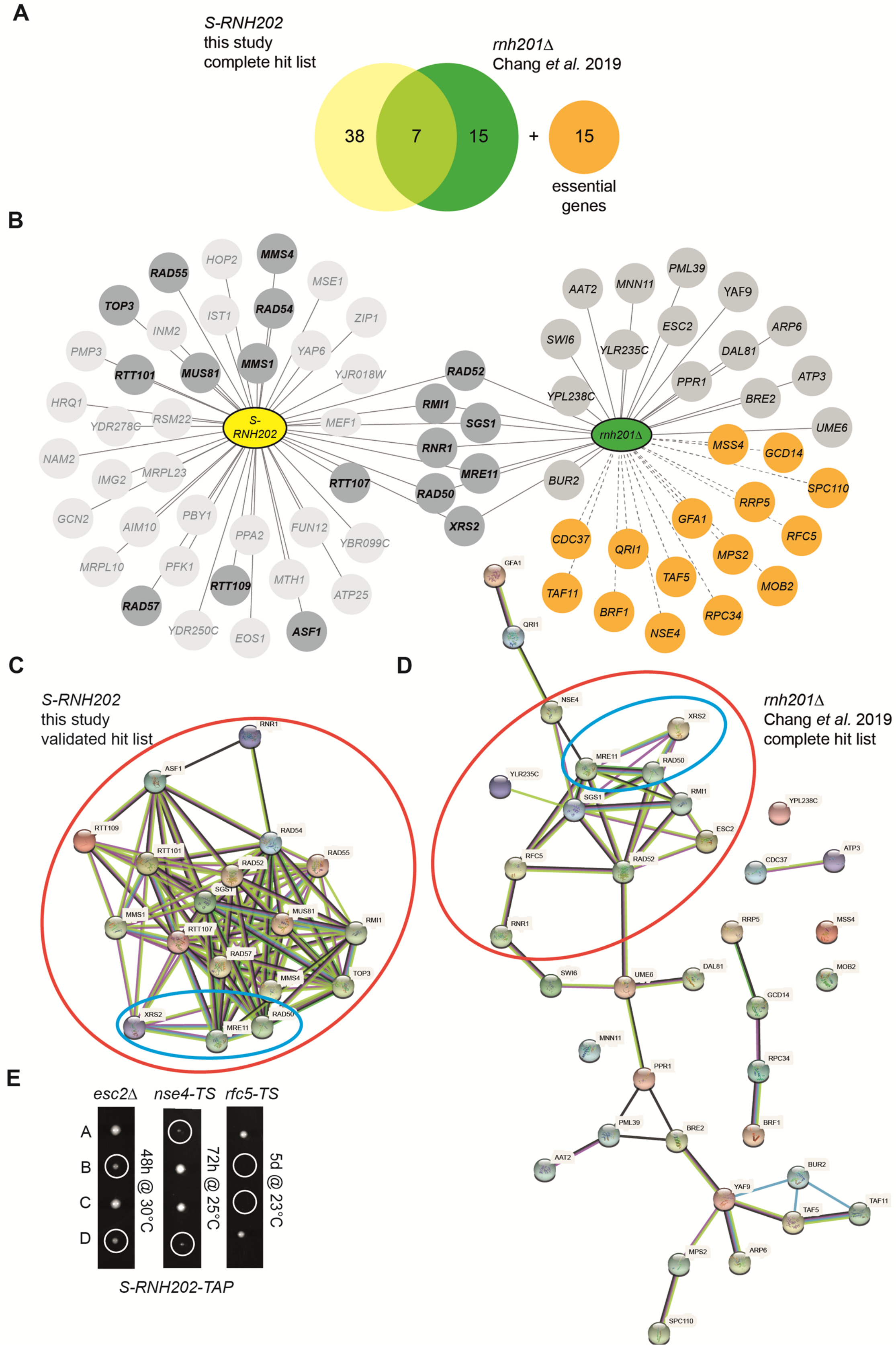
A
S-RNH202
this study
complete hit list
rnh201Δ
Chang et al. 2019
38
7
15
+
15
essential
genes
B
S-RNH202
rnh201Δ
HOP2
MMS4
RAD55
MSE1
TOP3
IST1
RAD54
ZIP1
INM2
PMP3
YAP6
MMS1
RTT101
MUS81
YJR018W
HRQ1
RSM22
YDR278C
MEF1
NAM2
MRPL23
IMG2
RTT107
GCN2
AIM10
PBY1
PPA2
FUN12
YBR099C
MRPL10
PFK1
MTH1
RTT109
ATP25
RAD57
YDR250C
EOS1
ASF1
RAD52
RMI1
SGS1
RNR1
MRE11
RAD50
XRS2
AAT2
MNN11
PML39
YAF9
ESC2
ARP6
SWI6
YLR235C
DAL81
PPR1
ATP3
YPL238C
BRE2
UME6
MSS4
GCD14
SPC110
BUR2
RRP5
GFA1
RFC5
CDC37
QRI1
MPS2
TAF5
MOB2
TAF11
BRF1
RPC34
NSE4
C
S-RNH202
this study
validated hit list
D
rnh201Δ
Chang et al. 2019
complete hit list
E
esc2Δ
nse4-TS
rfc5-TS
A
B
C
D
48h @ 30°C
72h @ 25°C
5d @ 23°C
S-RNH202-TAP

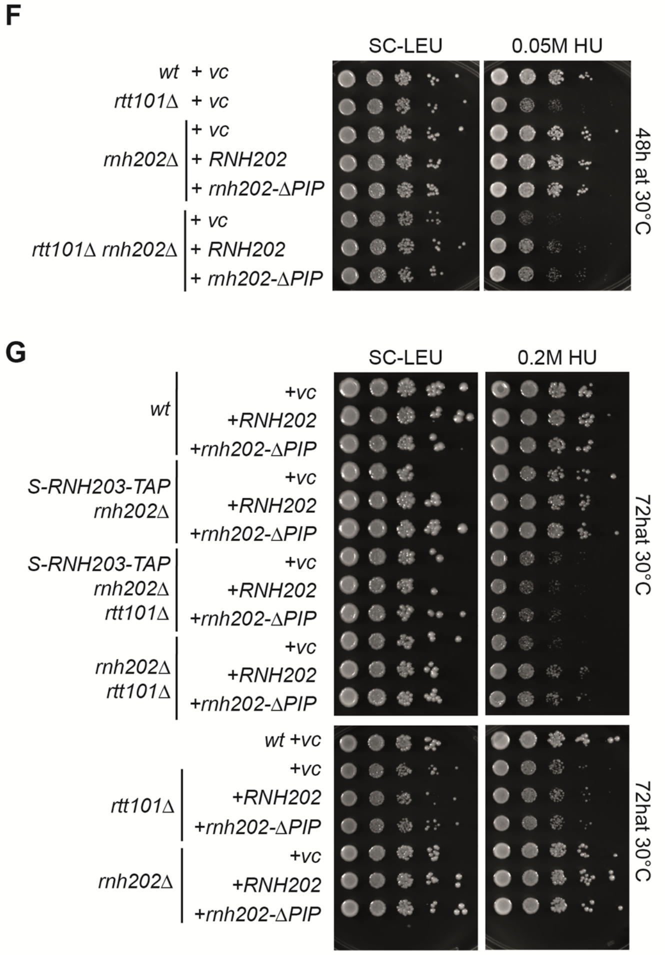

Supplementary Figure 2

Comparison of the synthetic sick genetic interactions in this study with the *S-RNH202* allele and a published RNase H2 deletion (*rnh201*Δ) dataset (Chang et al., 2019)**. (A)** Venn diagram shows that seven synthetic sick interactions are shared between *S-RNH202-TAP* and *rnh201*Δ. **(B)** String network of *S-RNH202-TAP* and *rnh201*Δ interactions (validated interactions dark grey). **(C)** String network among all validated synthetic sick interactions with *S-RNH202-TAP*. **(D)** String network among the complete hit list for *rnh201*Δ from both, the KO and TS collections, reveal a cluster of tightly interacting genes (red circle) that contains the MRX (*MRE11-RAD50-XRS2*) genes. This "MRX cluster" contains the seven genes shared between *S-RNH202-TAP* and *rnh201*Δ.: *MRE11, RAD50, XRS2, RNR1, RAD52, SGS1, RMI1.* In addition, the MRX cluster contains the following four genes: *YLR235C, RFC5, NSE4, ESC2*. *YLR235C* is a dubious open reading frame overlapping with the 3' end of the *TOP3* gene, hence is equivalent to a *TOP3* deletion. *ESC2*, *NSE*4, and *RFC5* could be validated. String networks were generated using the string-db.org web tool (Szklarczyk et al., 2021). **(E)** Tetrad dissection confirms the synthetic sick interactions of *S-RNH202-TAP* with *ESC2*, *NSE4,* and *RFC5*. **(F-G) The RNase H2**

PIP box motif has no effect on the synthetic sick genetic interaction between RNase H2 dysfunction and loss of *RTT101*. (F) Serial dilutions shows that the RNase H2 subunit lacking the C-terminal PIP box motif (*rnh202*- Δ*PIP*) can rescue viability of *rtt101*Δ *rnh202*Δ double mutant on HU to the same degree as the wild type *RNH202* subunit. **(G)** S phase restricted RNase H2 lacking the PIP box motif has the same negative genetic interaction with *rtt101*Δ as the wild type S phase restricted RNase H2.

Source data are provided as a Source Data file

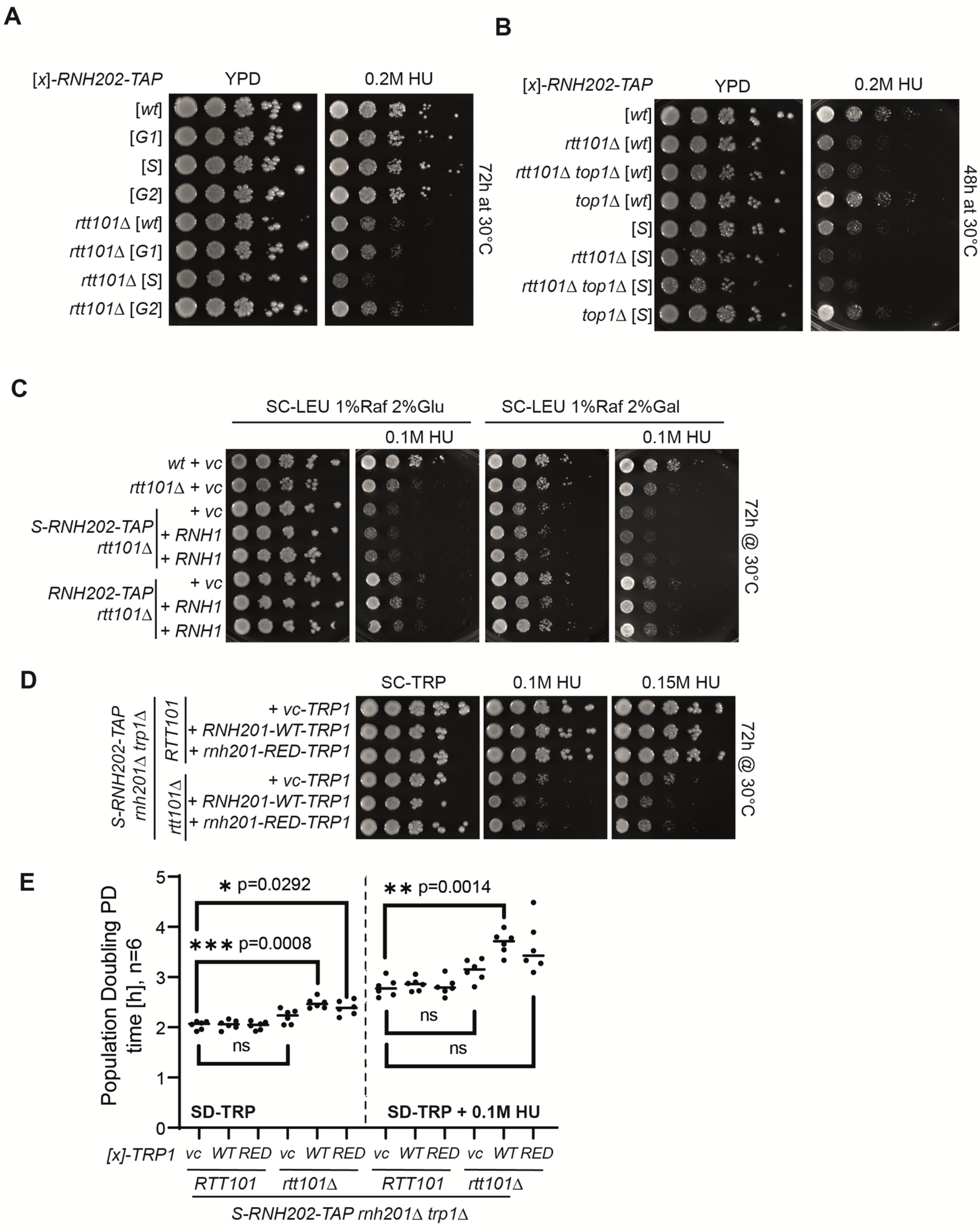

	S-RNH202 rnh201Δ trp1Δ			*rtt101Δ S-RNH202 rnh201Δ trp1Δ*		
	vc-TRP1	***RNH201-TRP1***	***rnh201-RED-TRP1***	***vc-TRP1***	***RNH201-TRP1***	***rnh201-RED-TRP1***
SC-TRP<br>PD [h] Median (n=6)	2.07	2.06	2.05	2.24	2.47	2.38
SD	0.08	0.09	0.08	0.14	0.10	0.14
SC-TRP **+ 0.1M HU**<br>PD [h] Median (n=6)	2.77	2.86	2.79	3.15	3.72	3,43
SD	0.18	0.13	0.18	0.21	0.23	0.51

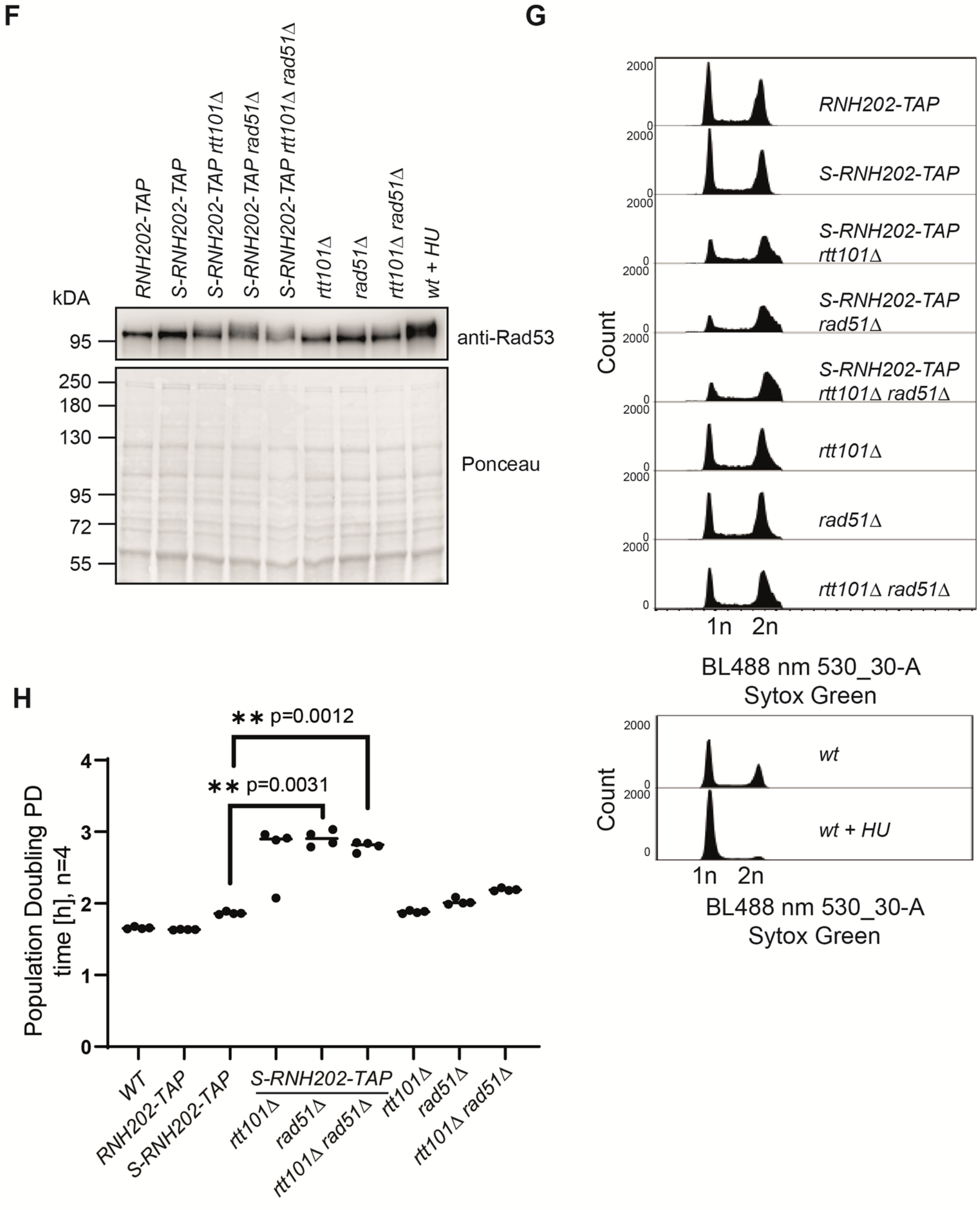

	wt	*RNH202-TAP*	*S-RNH202-TAP*	*S-RNH202-TAP rtt101Δ*	*S-RNH202-TAP rad51Δ*	*S-RNH202-TAP rad51Δ rtt101Δ*	*rtt101Δ*	*rad51Δ*	*rtt101Δ rad51Δ*
YPD									
PD [h] Median (n=4)	1.65	1.63	1.86	2.90	2.91	2.82	1.88	2.01	2.19
SD	0.02	0.00	0.02	0.42	0.11	0.07	0.02	0.04	0.02

Supplementary Figure 3

Loss of *RTT101* causes Top1-independent drop in viability in conditions of increased genomic rNMP hydrolysis. (A) The *rtt101Δ G1-RNH202* and *rtt101Δ G2-RNH202* double mutants are epistatic in terms of HU (hydroxy urea) sensitivity compared to *RNH202-TAP rtt101Δ*. The *S-RNH202 rtt101Δ* double mutant is very sick in the presence of HU. **(B)** Deletion of *TOP1* did not rescue of the toxicity of *S-RNH202-TAP rtt101Δ* in the presence of HU. **(C)** Spot assay using *RNH1* overexpression to test the

effect of R-loop removal in the *S-RNH202-TAP rtt101Δ* double mutant. **(D)** Spot assay to demonstrate that the rNMP-excision function of S phase restricted *RNH202* is causing the toxicity in the *S-RNH202-TAP rtt101Δ* double mutant. The *S-RNH202-TAP rnh201Δ rtt101Δ trp1Δ* quadruple mutant was transformed with the *pRS416-RNH201-WT-URA3* plasmid. Subsequently, the strains were co-transformed with *pRS413-vc-TRP1, pRS413-RNH201-WT-TRP1*, or *pRS413-rnh201-RED* plasmids. Then, we selected for loss of the *pRS416-RNH201-WT-URA3* plasmid in the presence of 5-FOA and spotted the resulting strains. **(E)** The same genotypes shown in S2D were grown in liquid SC-TRP medium to determine the population doubling (PD) time by growth curve analysis. We calculated the population doubling (PD) time from n=6 replicates. Scatter plot with line at median. Statistical analysis with GraphPadPrism8 (multiple comparison ANOVA test), ns=not significant. The table shows the PD in hours with the standard deviation (SD) of the replicates. **(F)** Western blot for Rad53 phospho-shift indicating the activated DNA damage checkpoint in the respective genotypes using samples from exponential growth phase. HU treated wild type served as positive control. **(G)** DNA profiles of SYTOX Green stained strains with the same genotypes shown in S2F. **(H)** Growth curve analysis of the indicated genotypes (same as in S2F, S2G) in liquid YPD medium. Population doubling (PD) time was calculated from n=4 replicates. The table shows the PD in hours with the standard deviation (SD). Note that there is one outlier in the replicates of the *S-RNH202 rtt101Δ* genotype leading to a lower significance. Scatter plot with line at median. Statistical analysis with GraphPadPrism8 (multiple comparison ANOVA test).

Source data are provided as a Source Data file.

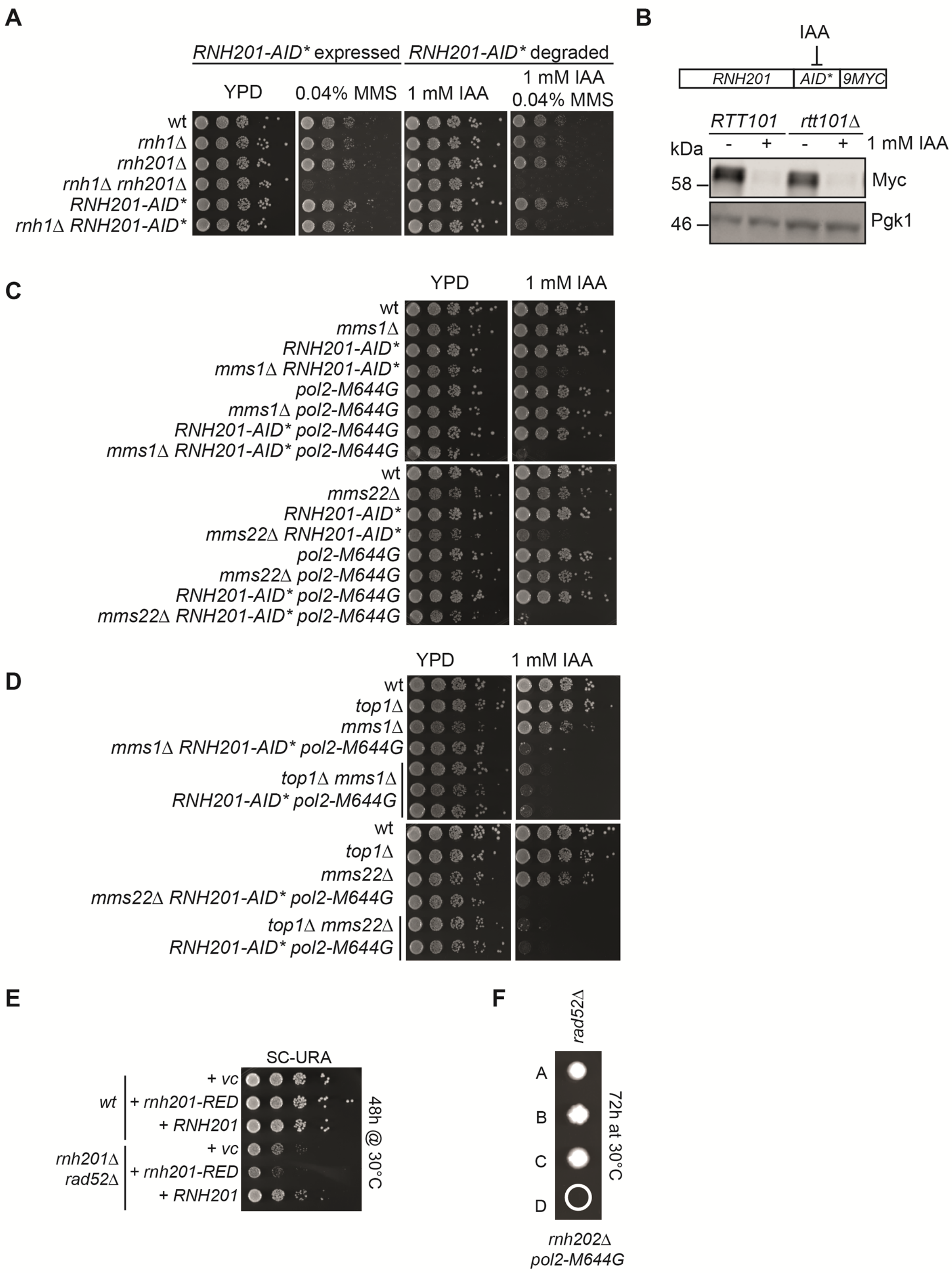
A
RNH201-AID* expressed
RNH201-AID* degraded
YPD
0.04% MMS
1 mM IAA
1 mM IAA 0.04% MMS
wt
rnh1∆
rnh201∆
rnh1∆ rnh201∆
RNH201-AID*
rnh1∆ RNH201-AID*
B
IAA
RNH201
AID*
9MYC
RTT101
rtt101∆
kDa
1 mM IAA
58
46
Myc
Pgk1
C
YPD
1 mM IAA
wt
mms1∆
RNH201-AID*
mms1∆ RNH201-AID*
pol2-M644G
mms1∆ pol2-M644G
RNH201-AID* pol2-M644G
mms1∆ RNH201-AID* pol2-M644G
wt
mms22∆
RNH201-AID*
mms22∆ RNH201-AID*
pol2-M644G
mms22∆ pol2-M644G
RNH201-AID* pol2-M644G
mms22∆ RNH201-AID* pol2-M644G
D
YPD
1 mM IAA
wt
top1∆
mms1∆
mms1∆ RNH201-AID* pol2-M644G
top1∆ mms1∆ RNH201-AID* pol2-M644G
wt
top1∆
mms22∆
mms22∆ RNH201-AID* pol2-M644G
top1∆ mms22∆ RNH201-AID* pol2-M644G
E
SC-URA
wt
+ vc
+ rnh201-RED
+ RNH201
rnh201∆ rad52∆
+ vc
+ rnh201-RED
+ RNH201
48h @ 30°C
F
rad52∆
A
B
C
D
72h at 30°C
rnh202∆ pol2-M644G

G

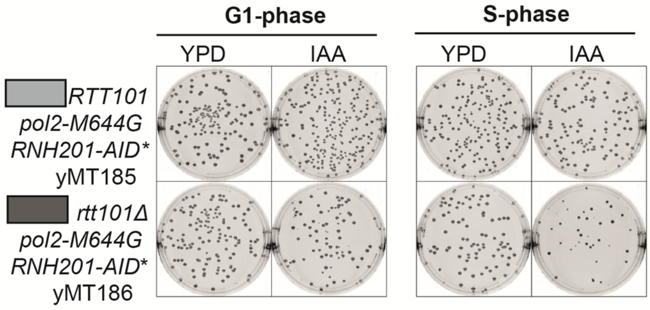
G1-phase
S-phase
YPD
IAA
YPD
IAA
RTT101
pol2-M644G
RNH201-AID*
yMT185
rtt101Δ
pol2-M644G
RNH201-AID*
yMT186

H

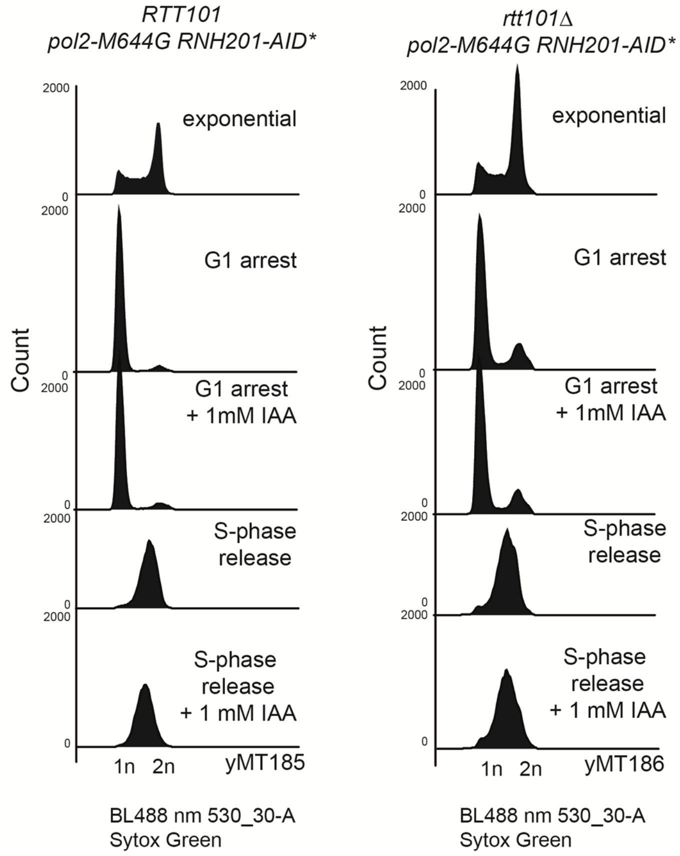
RTT101
pol2-M644G RNH201-AID*
rtt101Δ
pol2-M644G RNH201-AID*
Count
2000
0
exponential
G1 arrest
G1 arrest
+ 1mM IAA
S-phase
release
S-phase
release
+ 1 mM IAA
1n
2n
yMT185
yMT186
BL488 nm 530_30-A
Sytox Green

I

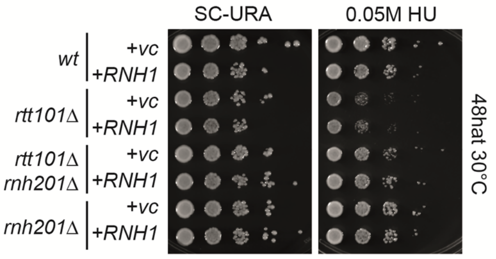
SC-URA
0.05M HU
wt
+vc
+RNH1
rtt101Δ
+vc
+RNH1
rtt101Δ
rnh201Δ
+vc
+RNH1
rnh201Δ
+vc
+RNH1
48hat 30°C

J

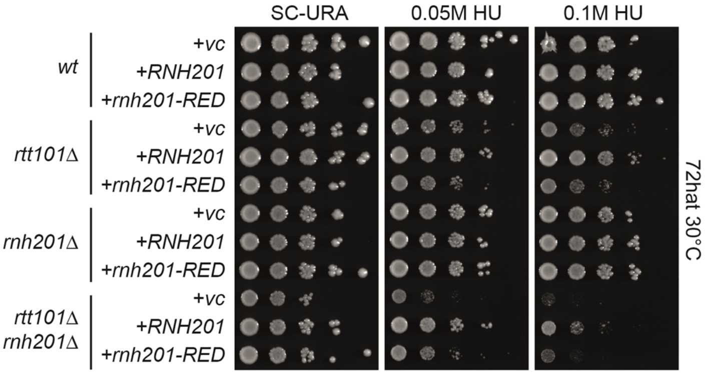
SC-URA
0.05M HU
0.1M HU
wt
+vc
+RNH201
+rnh201-RED
rtt101Δ
+vc
+RNH201
+rnh201-RED
rnh201Δ
+vc
+RNH201
+rnh201-RED
rtt101Δ
rnh201Δ
+vc
+RNH201
+rnh201-RED
72hat 30°C

K

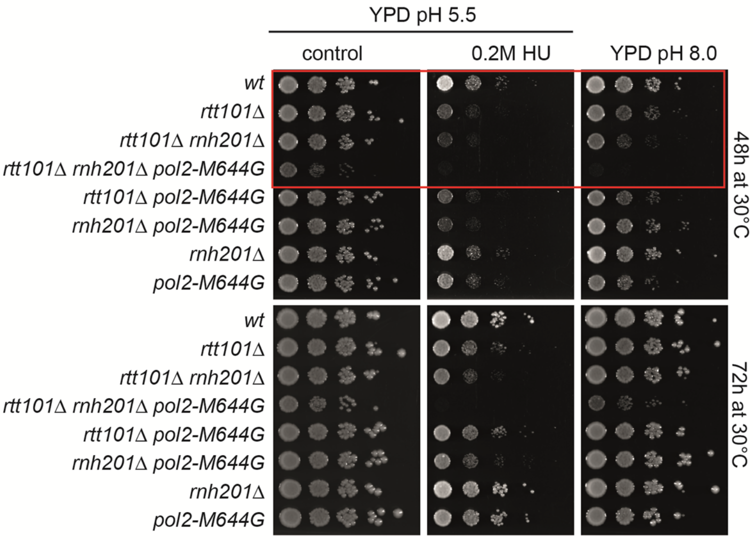
YPD pH 5.5
control
0.2M HU
YPD pH 8.0
wt
rtt101Δ
rtt101Δ rnh201Δ
rtt101Δ rnh201Δ pol2-M644G
rtt101Δ pol2-M644G
rnh201Δ pol2-M644G
rnh201Δ
pol2-M644G
48h at 30°C
wt
rtt101Δ
rtt101Δ rnh201Δ
rtt101Δ rnh201Δ pol2-M644G
rtt101Δ pol2-M644G
rnh201Δ pol2-M644G
rnh201Δ
pol2-M644G
72h at 30°C

Supplementary Figure 4

The synthetic sickness of the Rtt101$^{Mms1-Mms22}$ complex with RNase H2-deficiency is Top1-independent. (A) Functionality test of the *RNH201-AID** strain by spot assay. The *rnh1Δ rnh201Δ* double mutant is sensitive to MMS (methyl methane sulfonate) (Lazzaro et al., 2012). The depletion of *RNH201-AID** by IAA (auxin) in the *rnh1Δ* background impaired cell growth. **(B)** The auxin-inducible degron (AID*) tag results in proteasomal degradation of the fusion protein in the presence of auxin (Morawska & Ulrich, 2013). Western blot of exponential cells treated for 1 h with 1 mM IAA confirmed a robust degradation of the Rnh201-AID*-9Myc protein. **(C)** The depletion of *RNH201-AID** by auxin resulted in a synthetic sick growth phenotype with *mms1Δ*, and *mms1Δ*, which was amplified into a synthetic lethal phenotype when combined with high genomic rNMP load (*pol2-M644G*). **(D)** The synthetic lethality of the *pol2-M644G mms1Δ RNH201-AID** and the *pol2-M644G mms22 RNH201-AID** triple mutant on auxin plates was Top1-independent. Images were taken after 2 days of growth at 30°C. **(E)** Spot assay showing the effect of *RAD52* deletion in the RER-deficient RNase H2 mutant (*rnh201-RED*). **(F)** Tetrad dissection showed that *rnh202Δ pol2-M644G* double mutants require *RAD52* for survival. **(G-H) Colony formation assay representative images and DNA profiles. (G)** Images of representative agar plates from the colony formation assay. Summary of the data shown in Figure 3 F-H. **(H)** The DNA profiles of the strains used in the colony formation assay (G) and Figure 3 F-H were measured by flow cytometry. **(I-K)** Serial dilution control spottings for data shown in the main Figure 3 with all genotypes. **(I)** The serial dilution assay with *rtt101Δ* strains in RER-deficient *rnh201Δ* strain with RNase H1 overexpression. Control for spotting in Figure 3A. (J) Spot assay with *rtt101Δ* strains in the RER-deficient *rnh201Δ* and the rNMP accumulating *pol2-M644G* background with all control genotypes (selection of this data in the Figure 3B). **(K)** Control genotypes for the serial dilution shown in Figure 3I. The red box marks the part of the spotting that is identical with the selection in main Figure 3 panel I.

Source data are provided as a Source Data file

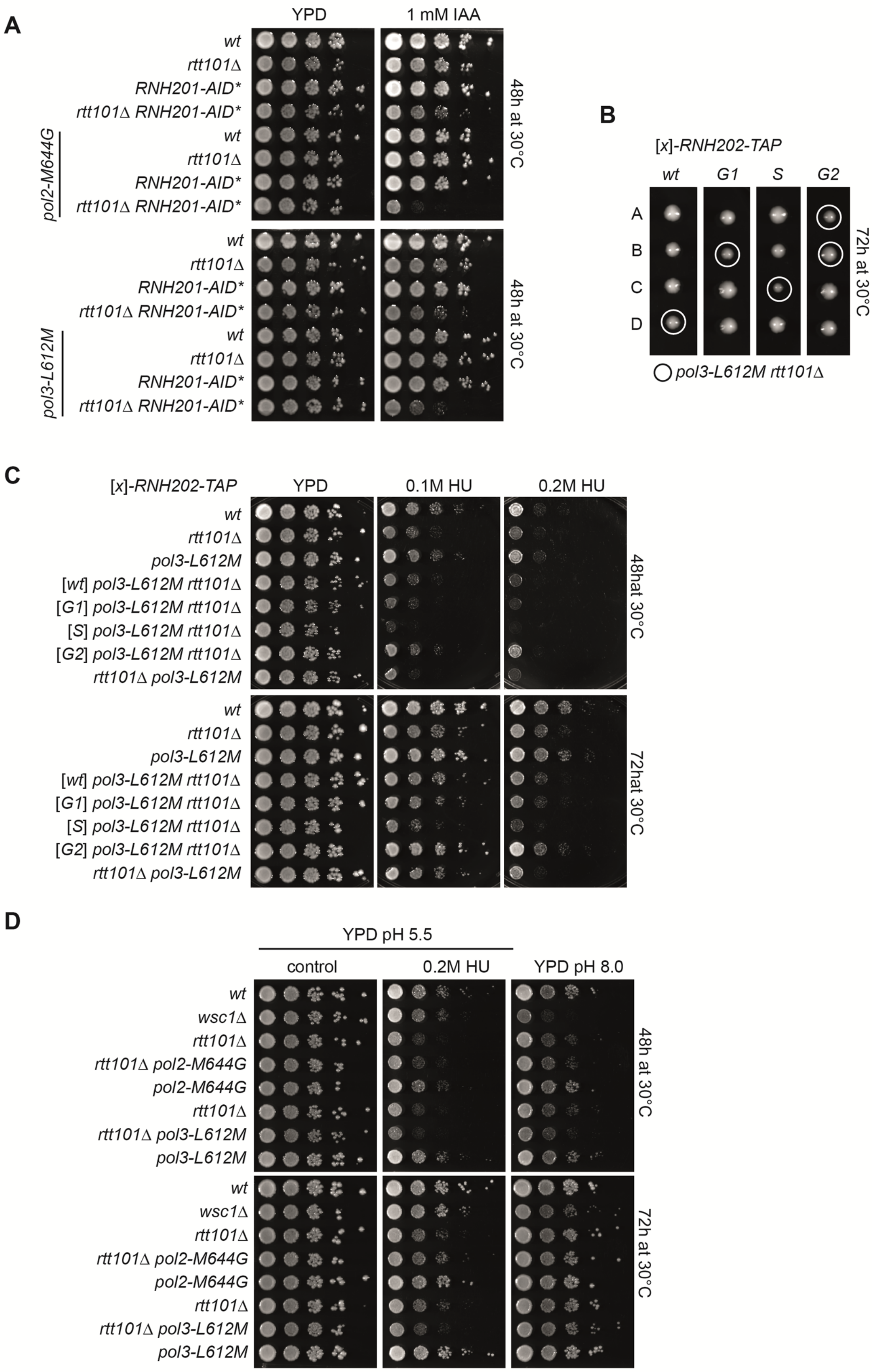

Supplementary Figure 5

The synthetic sickness of the Rtt101 with RNase H2-deficiency and S phase restricted RNase H2 is exacerbated in the presence of *pol3-L612M*. (A) Spot assay with the same genotypes as used in

Figure 3C compared to the corresponding genotypes having the *pol3-L612M* allele instead of the *pol2-M644G* allele. The spotting was done on a large agar plate so all 16 strains have been exposed to the exact same auxin (IAA) concentration and are hence comparable. Compared to the *pol2-M644G* mutator, the *pol3-L612M* mutator has a milder effect on the fitness of RER-deficient and *RTT101* deficient strains. This is in line with the 50% lesser rNMP incorporation rate of *POL3* compared to *POL2* (Williams et al., 2016). **(B)** Tetrad dissection shows that the synthetic sickness of *S-RNH202* allele and *rtt101*Δ is enhanced in the presence of *pol3-L612M* and still visible in terms of small colony after 72h outgrowth. Compare to Figure 2A and 2B. **(C)** As *S-RNH202 rtt101 pol2-M644G* strains were not viable after propagation from the tetrad, we could not compare but only do spot assays alone with the *pol3-L612M* containing strains that were all viable after the dissection. Spot assay showing that the *pol3-L612M* is the synthetic lethal with *S-RNH202 rtt101*Δ in the presence of hydroxy urea (HU). We noted a synthetic sickness between *rtt101*Δ and *pol3-L612M* in the presence of 0.2M HU and compared the mutator allele strains in a systematic way in panel D. **(D) C**omparison between *rtt101*Δ *pol2-M644G* and *rtt101*Δ *pol3-L612M* double mutants. The *rtt101*Δ *pol3-L612M* double mutant is slightly more pH sensitive at 48h and slightly more HU sensitive at 72h compared to the *rtt101*Δ *pol2-M644G* double mutant.

Source data are provided as a Source Data file.

Supplementary Figure 6

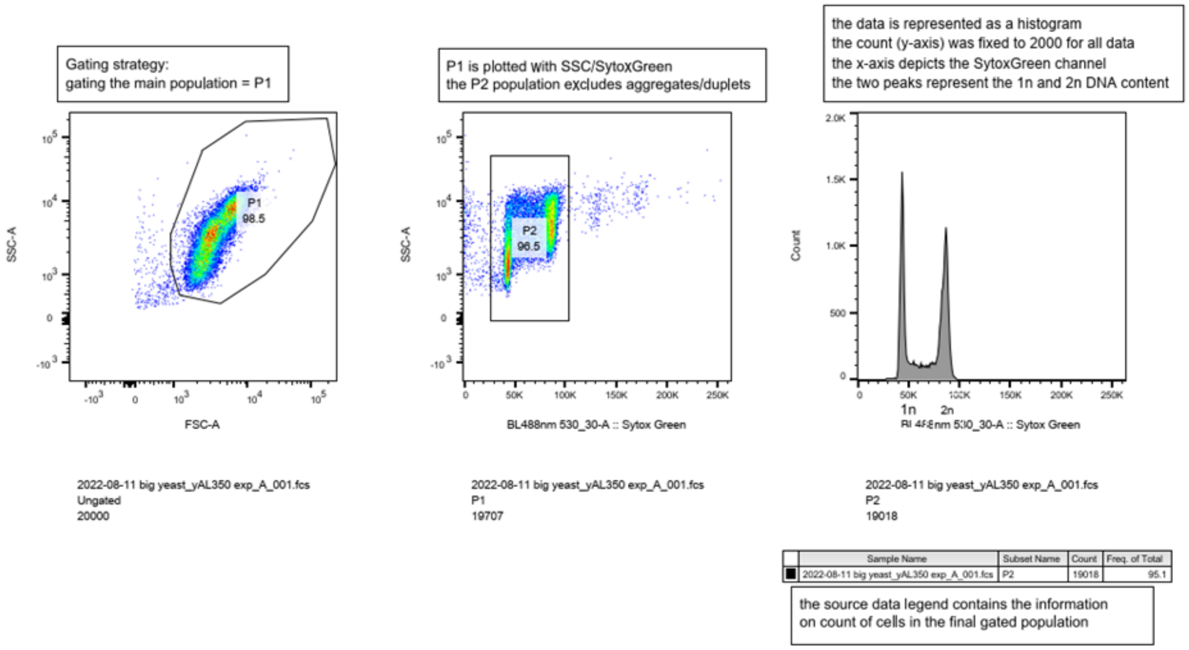

Gating strategy for the flow cytometry analysis of SYTOX Green stained and fixed cells as described in the methods.

Supplementary References

Chang, E. Y. C., Tsai, S., Aristizabal, M. J., Wells, J. P., Coulombe, Y., Busatto, F. F., Chan, Y. A., Kumar, A., Dan Zhu, Y., Wang, A. Y. H., Fournier, L. A., Hieter, P., Kobor, M. S., Masson, J. Y., & Stirling, P. C. (2019). MRE11-RAD50-NBS1 promotes Fanconi Anemia R-loop suppression at transcription–replication conflicts. *Nature Communications*, *10*(1). https://doi.org/10.1038/s41467-019-12271-w

Krogh, B. O., Llorente, B., Lam, A., & Symington, L. S. (2005). *Mutations in Mre11 Phosphoesterase Motif I That Impair Saccharomyces cerevisiae Mre11-Rad50-Xrs2 Complex Stability in Addition to Nuclease Activity*. *1570*(December), 1561–1570. https://doi.org/10.1534/genetics.105.049478

Lazzaro, F., Novarina, D., Amara, F., Watt, D. L., Stone, J. E., Costanzo, V., Burgers, P. M., Kunkel, T. A., Plevani, P., & Muzi-Falconi, M. (2012). RNase H and postreplication repair protect cells from ribonucleotides incorporated in DNA. *Molecular Cell*, *45*(1), 99–110. https://doi.org/10.1016/j.molcel.2011.12.019

Mankouri, H. W., & Hickson, I. D. (2006). *Top3 Processes Recombination Intermediates and Modulates Checkpoint Activity after DNA Damage* □. *17*(October), 4473–4483. https://doi.org/10.1091/mbc.E06

Morawska, M., & Ulrich, H. D. (2013). An expanded tool kit for the auxin-inducible degron system in budding yeast. *Yeast*, *30*(9), 341–351. https://doi.org/10.1002/yea.2967

Szklarczyk, D., Gable, A. L., Nastou, K. C., Lyon, D., Kirsch, R., Pyysalo, S., Doncheva, N. T., Legeay,

M., Fang, T., Bork, P., Jensen, L. J., & Mering, C. Von. (2021). *The STRING database in 2021 : customizable protein – protein networks , and functional characterization of user-uploaded gene / measurement sets*. *49*(November 2020), 605–612.

Williams, J. S., Lujan, S. A., & Kunkel, T. A. (2016). Processing ribonucleotides incorporated during eukaryotic DNA replication. *Nature Reviews Molecular Cell Biology*, *17*(6), 350–363. https://doi.org/10.1038/nrm.2016.37
