# Supplementary material for: Genetic requirements for repair of lesions caused by single genomic ribonucleotides in S phase
Source: Nat Commun. 2023 Mar 3;14:1227. doi: 10.1038/s41467-023-36866-6 (PMC9984532; doi:10.1038/s41467-023-36866-6)
Supplement: Supplementary file 2 — Description of Additional Supplementary Files [file 41467_2023_36866_MOESM2_ESM.pdf]

Description of Additional Supplementary Files

File Name: Supplementary Data 1
Description: SGA screen hit (Figure 1) evaluation by manual dissection. Representative tetrad dissection images in Figure S1. The exclusive G2- RNH202 allele hits, YLR236 and FYV10 were not verified but greyed out in the string network in Figure 1G as they were not genetically interacting with the S-RNH202 allele.

File Name: Supplementary Data 2
Description: : Yeast strains used in this study.

File Name: Supplementary Data 3
Description: Plasmids and oligonucleotides used in this study.

File Name: Supplementary Data 4
Description: Materials (Antibodies, Enzymes, Chemicals) used in this study.

File Name: Supplementary Data 5
Description: RNH202_cell cycle KO screen data.
