# Supplementary material for: Genetic requirements for repair of lesions caused by single genomic ribonucleotides in S phase
Source: Nat Commun. 2023 Mar 3;14:1227. doi: 10.1038/s41467-023-36866-6 (PMC9984532; doi:10.1038/s41467-023-36866-6)
Supplement: Supplementary file 8 — Reporting Summary [file 41467_2023_36866_MOESM8_ESM.pdf]

Corresponding author(s): Natalie Schindler, Brian Luke

Last updated by author(s): Jan 24, 2023

Reporting Summary

Nature Portfolio wishes to improve the reproducibility of the work that we publish. This form provides structure for consistency and transparency in reporting. For further information on Nature Portfolio policies, see our Editorial Policies and the Editorial Policy Checklist.

Statistics

For all statistical analyses, confirm that the following items are present in the figure legend, table legend, main text, or Methods section.

n/a	Confirmed	
☐	☒	The exact sample size (*n*) for each experimental group/condition, given as a discrete number and unit of measurement
☐	☒	A statement on whether measurements were taken from distinct samples or whether the same sample was measured repeatedly
☐	☒	The statistical test(s) used AND whether they are one- or two-sided<br>*Only common tests should be described solely by name; describe more complex techniques in the Methods section.*
☐	☒	A description of all covariates tested
☐	☒	A description of any assumptions or corrections, such as tests of normality and adjustment for multiple comparisons
☐	☒	A full description of the statistical parameters including central tendency (e.g. means) or other basic estimates (e.g. regression coefficient) AND variation (e.g. standard deviation) or associated estimates of uncertainty (e.g. confidence intervals)
☐	☒	For null hypothesis testing, the test statistic (e.g. *F*, *t*, *r*) with confidence intervals, effect sizes, degrees of freedom and *P* value noted<br>*Give P values as exact values whenever suitable.*
☒	☐	For Bayesian analysis, information on the choice of priors and Markov chain Monte Carlo settings
☒	☐	For hierarchical and complex designs, identification of the appropriate level for tests and full reporting of outcomes
☒	☐	Estimates of effect sizes (e.g. Cohen's *d*, Pearson's *r*), indicating how they were calculated

Our web collection on statistics for biologists contains articles on many of the points above.

Software and code

Policy information about availability of computer code

Data collection	SGA screen data evaluation: Imaging with the Singer PhenoBooth colony imager.<br>Flow cytometry: Data were collected using the BD LSRFortessa flow cytometer (BD Biosciences) using the BD FACSDiva software (v9.0.1)
Data analysis	SGA screen data evaluation: Data analysis was performed in R (R Core Team (2021). R: A language and environment for statistical computing. http://www.R-project.org/) as detailed in the R vignette (S6 HTML).<br>Flow cytometry: Data analysis was performed with FlowJo (v10.8.0).<br>Canavanine mutagenesis assay: the data was plotted as the median with 95% Confidence interval using the GraphPad PRIMS8 software.<br>Plating assay: the statistical analysis and plot generation was performed using GraphPad PRISM8 software.<br>Alkaline gel electrophoresis: densiometric analysis was performed using Fiji/ImageJ and plots were generated using GraphPad PRISM8 software.

For manuscripts utilizing custom algorithms or software that are central to the research but not yet described in published literature, software must be made available to editors and reviewers. We strongly encourage code deposition in a community repository (e.g. GitHub). See the Nature Portfolio guidelines for submitting code & software for further information.

Data

Policy information about availability of data

All manuscripts must include a data availability statement. This statement should provide the following information, where applicable:

- Accession codes, unique identifiers, or web links for publicly available datasets
- A description of any restrictions on data availability
- For clinical datasets or third party data, please ensure that the statement adheres to our policy

The SGA screen data is provided as Supplementary Data S1/S7 with gene names and identifiers. All yeast strains (Supplementary Data S2), plasmids and oligonucleotides (Supplementary Data S3) are accessible and the gene names according to Saccharomyces Genome Database (www.yeastgenome.org) are given. All materials are listed in Supplementary Data S4 and the relevant raw data like uncropped images of gels and blots) are provided in the numerical data Supplementary Data S5. The R vignette used to analyse the SGA screen data is provided as HTML file Supplementary Data S6..

Human research participants

Policy information about studies involving human research participants and Sex and Gender in Research.

Reporting on sex and gender	no human research was done, study is in yeast
Population characteristics	no human research was done, study is in yeast
Recruitment	no human research was done, study is in yeast
Ethics oversight	no human research was done, study is in yeast

Note that full information on the approval of the study protocol must also be provided in the manuscript.

Field-specific reporting

Please select the one below that is the best fit for your research. If you are not sure, read the appropriate sections before making your selection.

☒ Life sciences ☐ Behavioural & social sciences ☐ Ecological, evolutionary & environmental sciences

For a reference copy of the document with all sections, see nature.com/documents/nr-reporting-summary-flat.pdf

Life sciences study design

All studies must disclose on these points even when the disclosure is negative.

Sample size	The sample sizes, number of replicates and choice of statistical methods were determined based on previous experience by the co-authors, literature references, and the common practice in the field. At least 2 independent replicates were performed. Of plasmids and strains, multiple clones were tested. For non-quantitative Western blot and alkaline gel electrophoresis, two replicates were analysed.
Data exclusions	SGA screen analysis: False positives and linked genes have been excluded in the final analysis (greyed out in Figure 1).
Replication	We verified the reproducibility by confirming the SGA screen candidates by manual tetrad dissection of crossed that were independently done from scratch. All findings in the manuscript were reproducible in at least 2 replicates.
Randomization	Not applicable to our types of experiments as no case studies were done. The complete study was done using yeast.
Blinding	The SGA screen was performed blinded as the executer did evaluate the images of colonies under the criteria before assigning the gene identifer to the areas on the plates. Hence, the evaluation was not prone to experimentalist bias. The tetrad dissections were also protected from bias as the genotyping and scoring occurs after colony outgrowth. The other types of data, e.g. Western blot, prevent blinding as it is important for presentation purpose to load gels in order.

Reporting for specific materials, systems and methods

We require information from authors about some types of materials, experimental systems and methods used in many studies. Here, indicate whether each material, system or method listed is relevant to your study. If you are not sure if a list item applies to your research, read the appropriate section before selecting a response.

Materials & experimental systems

n/a	Involved in the study
☐	☒ Antibodies
☒	☐ Eukaryotic cell lines
☒	☐ Palaeontology and archaeology
☒	☐ Animals and other organisms
☒	☐ Clinical data
☒	☐ Dual use research of concern

Methods

n/a	Involved in the study
☒	☐ ChIP-seq
☐	☒ Flow cytometry
☒	☐ MRI-based neuroimaging

Antibodies

Antibodies used

all antibodies are listed in Supplementary Data S4. Here the name (dilution, company, order ID): anti-Rad53 antibody (1:1000, Abcam, Cat#ab166859), Rabbit Peroxidase Anti-Peroxidase soluble complex (1:2000, Sigma-Aldrich, Cat#P1291), Mouse monoclonal anti-Phosphoglycerate Kinase 1 (22C5D8) (1:10.000, Invitrogen Cat#459250), Mouse anti-Myc-Tag (9811) (1:1000, Cell Signaling, Cat#2276S), Goat Immun-Star anti-mouse (GAM)-HRP conjugate (1:3000, Bio-Rad Cat#170-5047), Goat Immun-Star anti-rabbit (GAR)-HRP conjugate (1:3000, Bio-Rad, Cat#170-5046)

Validation

the antibodies used were validated by the manufacturers, see here:
https://www.abcam.com/rad53-antibody-el7e1-ab166859.html
https://www.sigmaaldrich.com/DE/en/product/sigma/p1291
https://www.thermofisher.com/antibody/product/PGK1-Antibody-clone-22C5D8-Monoclonal/459250
https://www.cellsignal.com/products/primary-antibodies/myc-tag-9b11-mouse-mab/2276
https://www.bio-rad.com/de-de/sku/1705047-immun-star-goat-anti-mouse-gam-hrp-conjugate?ID=1705047
https://www.bio-rad.com/de-de/sku/1705046-immun-star-goat-anti-rabbit-gar-hrp-conjugate?ID=1705046

Flow Cytometry

Plots

Confirm that:

☒ The axis labels state the marker and fluorochrome used (e.g. CD4-FITC).

☒ The axis scales are clearly visible. Include numbers along axes only for bottom left plot of group (a 'group' is an analysis of identical markers).

☒ All plots are contour plots with outliers or pseudocolor plots.

☒ A numerical value for number of cells or percentage (with statistics) is provided.

Methodology

Sample preparation

For DNA content analysis, cells were fixed in 70% ethanol overnight and then treated with 0.25 mg/ml DNase- and Protease-free RNase A (ThermoFisher Scientific, 10753721) at 37°C for 2h and Proteinase K (Biofroxx, 1151ML010) at 50°C for 2h in 50 mM Tris-HCl pH7.5 buffer. The cell suspension was sonified using a Branson sonifier 450 for 5 sec with output control 1 and duty cycle constant. Then, cells were stained with a final concentration of 2.4 µM SYTOX Green nucleic acid stain (ThermoFisher Scientific, 1076273). For cell viability analysis, cells were collected and the cell pellet was washed with 50 mM Tris pH 7.5 and resuspended in 1 ml 50 mM Tris pH 7.5 containing 0.5 µM SYTOX Green. Measurement and analysis were the same as for the DNA content analysis except for doublet exclusion, which was done in the SSC-A vs. W channel. As a control sample for dead cells, controls were incubated at 95°C for 15 min and subjected to the described protocol.

Instrument

BD LSRFortessa flow cytometer (BD Biosciences)

Software

BD FACSDiva software (v9.0.1), FlowJo (v10.8.0)

Cell population abundance

With low flow rate, 20,000 events were recorded.

Gating strategy

Analysis was performed with FlowJo (v10.8.0) using the following gating strategy: From the main population in FSC-A vs. SSC-A, doublets were excluded in the Sytox-Green A vs. W channel, and DNA content was assessed in the histogram of the Sytox-Green-A channel (Ex 488nm, 530/30BP). The gating strategy is shown in Supplemenary Figure S6.
The plots in the main figures are depicted as histograms as common for DNA profiles used for cell cycle analysis; dot blots and the number of cells is provided in the source data (Supplementary Data S5).

☒ Tick this box to confirm that a figure exemplifying the gating strategy is provided in the Supplementary Information.
